# N4BP3 facilitates NOD2-MAPK/NF-κB pathway in inflammatory bowel disease through mediating K63-linked RIPK2 ubiquitination

**DOI:** 10.1038/s41420-024-02213-x

**Published:** 2024-10-17

**Authors:** Wang Jiang, Yan Zhao, Min Han, Jiafan Xu, Kun Chen, Yi Liang, Jie Yin, Jinyue Hu, Yueming Shen

**Affiliations:** 1 Department of Digestive Diseases, The Affiliated Changsha Central Hospital, Hengyang Medical School, University of South China, 161 Shaoshan Road, Changsha, 410000 China; 2 Department of Pathology, The Affiliated Changsha Central Hospital, Hengyang Medical School, University of South China, 161 Shaoshan Road, Changsha, 410000 China; 3 Department of Cardiovascular Diseases, The Affiliated Changsha Central Hospital, Hengyang Medical School, University of South China, 161 Shaoshan Road, Changsha, 410000 China; 4 Department of Orthopaedics, The Affiliated Changsha Central Hospital, Hengyang Medical School, University of South China, Changsha, Hunan China; 5 Department of Gastrointestinal Surgery, The Second Affiliated Hospital, Hengyang Medical School, University of South China, 35 Jiefang Road, Hengyang, 421000 China; 6 Medical Research Center, The Affiliated Changsha Central Hospital, Hengyang Medical School, University of South China, Changsha, 410004 China

**Keywords:** NOD-like receptors, Inflammatory bowel disease, NOD-like receptors

## Abstract

The NOD2 signaling pathway, which plays an important role in the mechanisms of inflammatory bowel disease (IBD) development, has been closely associated with ubiquitination. It was revealed in this study that NOD2 receptor activation could obviously affect the expression of 19 ubiquitination-related genes, with N4BP3 being the most prominently expressed and upregulated. In addition, N4BP3 knockdown was found to reduce the mRNA levels of MDP-induced inflammatory factors, while N4BP3 overexpression elevated their mRNA levels as well as the levels of phospho-ERK1/2, phospho-JNK, phospho-P38 and phospho-NF-κB P65 proteins. Immunoprecipitation tests showed that N4BP3 could pull down RIPK2 and promote its K63-linked ubiquitination. In human tissue specimen assays and mouse experiments, we found that the expression of N4BP3 was significantly elevated in Crohn’s disease (CD) patients and IBD mice, and N4BP3 knockdown reduced the dextran sulfate sodium-induced pathological score and the expression of inflammatory factors in the mouse colon tissue. In conclusion, N4BP3 is able to interact with RIPK2 and promote its K63-linked ubiquitination, to further promote the NOD2-MAPK/NF-κB pathway, thereby increasing promoting the release of inflammation factors and the degree of IBD inflammation.

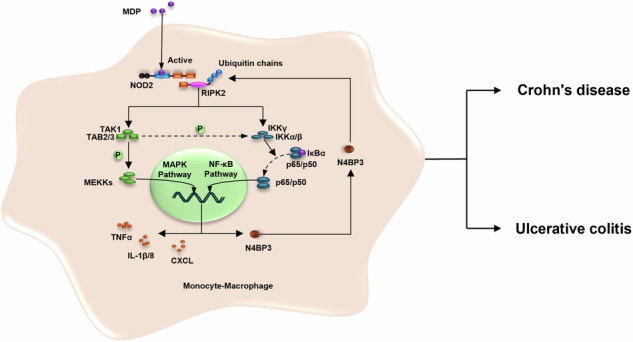

## Introduction

Inflammatory bowel disease (IBD) refers to a group of chronic non-specific inflammatory diseases of the intestine, including Crohn’s disease (CD) and ulcerative colitis (UC) [1]. The pathogenic mechanism of IBD, though having not been fully clarified, is currently believed to be related to environmental, genetic, immune, and intestinal microbiota factors [2], among which intestinal immune imbalance was been revealed to play an important role in the pathogenesis of IBD [3, 4]. On the other hand, pattern recognition receptors (PRRs), mainly including toll like receptor (TLR), retinoic acid inducible gene-1 like receptor (RLR), and NOD like receptor (NLR) [5], were found to play roles in monitoring pathogens and initiating the body’s inflammatory responses in the inflammatory immunity [6].

Ubiquitination is a critical mode of post-translational modification of proteins, with its main effects including protein degradation and functional regulation [7]. This biological process involves the participation of and is regulated by the ubiquitin-proteasome system (UPS) [8]. Ubiquitin is a globular protein composed of 76 amino acids [9]; it can form a ubiquitin chain by connecting its seven lysine residues (K6, K11, K27, K29, K33, K48, and K63) or the first methionine (M1) [10]. Ubiquitinating enzymes can be classified into ubiquitin-activating enzymes (E1), ubiquitin-conjugating enzymes (E2), and ubiquitin ligases (E3) [11]. More specifically, E3, which exerts crucial effects due to its substrate specificity, can be further divided into the HECT domain family, the RING domain family, and the RBR domain family [11]. Deubiquitinating enzymes can be classified into seven families, namely ubiquitin-specific proteases (USPs), Otubaims (OTUs), ubiquitin carboxyl-terminal hydrolases (UCHs), Machado-Joseph disease protein domain proteases (MJDs), JAB1/MPN domain-associated metallopeptidases (JAMMs), monocyte chemotactic protein-induced proteases (MCPIPs), and the MINDY family [11]. In the ubiquitination process, E1 is responsible for activating free ubiquitin and transferring it to E2, where activated ubiquitin forms a complex with E3 and the substrate protein, thereby causing ubiquitination modification of the substrate protein; consequently, the substrate protein is activated or is recognized and degraded by 26S proteasome [12]. Deubiquitinating enzymes may exert a certain antagonistic effect by removing ubiquitin [13].

Ubiquitination modification can regulate the immune inflammatory network so as to affect the occurrence and development of inflammation and is therefore expected to become a new treatment target for IBD. The TRIM26-mediated polyubiquitination of TAB1 can promote the activation of TAK1 as well as the subsequent activation of NF-κB and MAPK signaling pathways in macrophages, so as to exert a pro-inflammatory effect in sodium dextran sulfate (DSS)-induced colitis [14]. RNF183 was revealed to promote the NF-κB signaling-mediated intestinal inflammation by increasing the ubiquitination degradation of IκB [15]. TRIM31 can inhibit NLRP3 activation by promoting the NLRP3 inflammasome polyubiquitination and proteasome degradation, thereby alleviating DSS-induced colitis [16]. TRIM58 can regulate TLR2 in bone marrow cells through ubiquitination and inhibit intestinal inflammation by terminating the excessive activation of the NF-κB/AP-2 signaling pathway that is induced by TLR1 [17]. RNF8 ubiquitination can degrade AKT1 and inhibit the Akt/mTOR signaling pathway to enhance autophagy, which plays a role in reducing intestinal inflammation [18]. The deubiquitinating enzyme USP16 can promote the interaction between IKKβ and p105 and phosphorylate p105 by selectively removing K33-linked polyubiquitin chains from IKKβ, thereby activating the NF-κB signaling pathway and promoting intestinal inflammation [19]. The deubiquitinating enzyme CYLD was found to negatively regulate the activation of inflammasomes and IL-18 production in intestinal epithelial cells by mediating the deubiquitination of NLRP6, which consequently inhibits intestinal inflammation [20].

To date, there is still a lack of systematic research on the relationship between the NOD2 receptor pathway and ubiquitination. In this paper, we induced activation of NOD2 receptor in monocytes (THP-1 cells) using Muramyl Dipeptide (MDP), and conducted transcriptomics tests by taking untreated THP-1 cells as the control group. The results revealed differential expression between the two groups in 19 genes, including N4BP3, ZC3H12A, RNF144B, RNF122, RNF150, TRIM36, TRIM13, CBX4, CBX8, MINDY3, CYLD, TIFA, UBE2B, BIRC3, TNFAIP3, TRAF3, DTX2, KLHL21, and USP2. Moreover, the results of fluorescence quantitative PCR test highlighted that the expression of N4BP3 exhibited the most significant difference among the 19 genes.

N4BP3, the NEDD4 binding protein 3, is a member of the Fezzin family, consisting of a C-terminal Fez1 domain, a central curly domain, and a highly conserved PY domain that binds to NEDD4 [21]. On the whole, there is currently limited research on N4BP3. In earlier studies, N4BP3 was demonstrated to play a crucial role in axonal and dendritic branching [22], and was found to be expressed in the early anterior nerve tissue of African clawed frogs, and when absent, deformities were observed in the cartilage structures of the eyes, brain, and skull [21]. In terms of tumors, N4BP3 is able to promote the expression of STAT2 by interacting with KAT3B, thereby promoting the angiogenesis in hepatocellular carcinoma, making N4BP3 a potential therapeutic target for hepatocellular carcinoma [23]. Besides, N4BP3 may also play a role in promoting the metastasis of breast cancer by intensifying the ubiquitination and degradation of E-cadherin [24]. In terms of inflammation and immunity, N4BP3 overexpression can enhance the activation of SeV-induced ISRE and interferon β promoters, while N4BP3 knockout can inhibit the SeV-induced transcription of downstream antiviral genes [25]. To date, no studies on the relationship between N4BP3 and IBD have been reported, but we were lucky enough to find a correlation between them, and we aimed to clarify the role of N4BP3 in IBD as well as the underlying mechanisms in this paper.

## Results

### N4BP3 expression is significantly increased after NOD2 activation

**Fig. 1:**
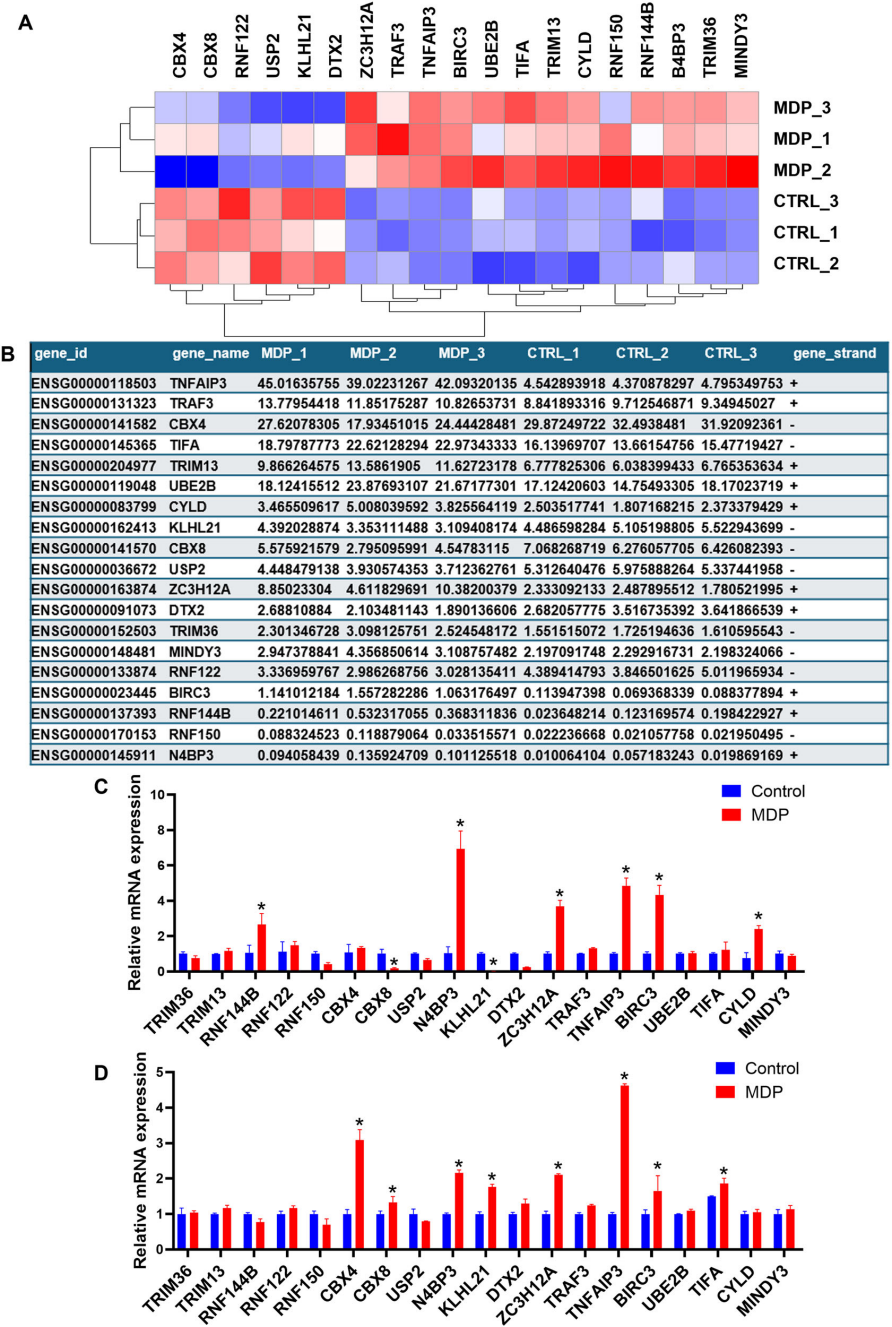
RNA-seq of ubiquitination-related genes that are related to the NOD2 receptor pathway. **A**, **B** The RNA-seq sequencing results. MDP refers to the experiment group, in which the THP-1 cells were induced by MDP (10 μg/mL). CTRL refers to the control group, in which the THP-1 cells were induced by an equal amount of culture medium. **C** The mRNA expression of different genes after NOD2 receptor activation in THP-1 cells. MDP refers to the experiment group, in which the THP-1 cells were induced by MDP (10 μg/mL). Control refers to the control group, in which the THP-1 cells were induced by an equal amount of culture medium. **D** The mRNA expression of different genes after NOD2 receptor activation in HCT116 cells. *p < 0.05, indicates a statistically significant difference as compared to the control group.

To screen for ubiquitination genes that are related to the NOD2 receptor pathway, we conducted a comparative experiment between the THP-1 cells induced by MDP (10 μg/mL) for NOD2 receptor activation [26–28] (experimental group) and THP-1 cells with an equal amount of culture medium (control group). Transcriptome sequencing was conducted using the Illumina sequencing platform to obtain RNA-Seq results for the purpose of enriching genes with ubiquitinating and deubiquitinating effects. The results showed that there were 19 ubiquitination-related genes presenting with differential expression between the two groups, including N4BP3, ZC3H12A, RNF144B, RNF122, RNF150, TRIM36, TRIM13, CBX4, CBX8, MINDY3, CYLD, TIFA, UBE2B, BIRC3, TNFAIP3, TRAF3, DTX2, KLHL21, and USP2 (Fig. 1A, B).

Then, RT-qPCR was performed to detect the mRNA expression of the 19 genes identified above. After activating the NOD2 receptor in THP-1 cells with MDP, the mRNA expression levels of N4BP3, TNFAIP3, BIRC3, ZC3H12A, RNF144B, and CYLD were significantly upregulated (*p* < 0.05), with the mRNA expression of N4BP3 presenting with the most significant difference (Fig. 1C); the mRNA expression levels of CBX8 and KLHL21 were significantly downregulated (*p* < 0.05) (Fig. 1C). Moreover, when taking HCT116 cells induced by MDP (25 μg/mL) for 4 h for NOD2 receptor activation [29] as the experimental group and HCT116 cells with an equal amount of culture medium as the control group, the RT-qPCR results showed that the mRNA expression levels of N4BP3, ZC3H12A, TNFAIP3, TIFA, CBX4, and KLHL21 were significantly upregulated (*p* < 0.05) (Fig. 1D).

**Fig. 2:**
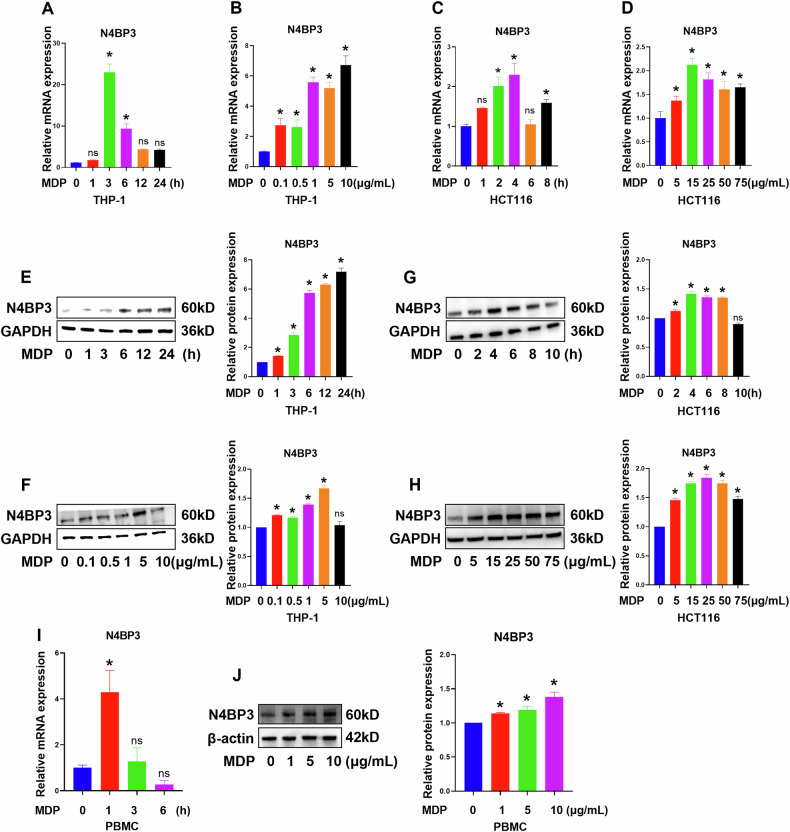
Expression of N4BP3 in THP-1/HCT116 cells induced by MDP. **A** The expression of N4BP3 mRNA in THP-1 cells induced by MDP (10 μg/mL) for different times. **B** The expression of N4BP3 mRNA in THP-1 cells induced by MDP at different concentrations. **C** The expression of N4BP3 mRNA in HCT116 cells induced by MDP (25 μg/mL) for different times. **D** The expression of N4BP3 mRNA in HCT116 cells induced by MDP at different concentrations. **E** The expression of N4BP3 protein in THP-1 cells induced by MDP (10 μg/mL) for different times. **F** The expression of N4BP3 protein in THP-1 cells induced by MDP at different concentrations. **G** The expression of N4BP3 protein in HCT116 cells induced by MDP (25 μg/mL) for different times. **H** The expression of N4BP3 protein in HCT116 cells induced by MDP at different concentrations. **I** The expression of N4BP3 mRNA in PBMCs induced by MDP (10 μg/mL) for different times. **J** The expression of N4BP3 protein in PBMCs induced by MDP at different concentrations. *p < 0.05, indicates a statistically significant difference as compared to the control group.

To further verify the changes in N4BP3 expression after NOD2 receptor activation, we induced THP-1 cells with MDP (10 μg/mL) for different durations (0 h, 1 h, 3 h, 6 h, 12 h, 24 h), and detected the mRNA and protein expression levels of N4BP3. The results showed that, as the induction time increased, both the mRNA and protein levels of N4BP3 were increased (Fig. 2A, E); more specifically, the mRNA level was significantly increased at 3 h and 6 h (*p* < 0.05), with the highest expression observed at 3 h (Fig. 2A). Then, we induced THP-1 cells with MDP at different concentrations (0, 0.1, 0.5, 1, 5, 10 μg/mL) for 3 h, and detected the mRNA and protein expression levels of N4BP3. The results showed that, compared with 0 μg/mL MDP, the mRNA and protein levels of N4BP3 were increased after induction with MDP at all other concentrations (Fig. 2B, F); more specifically, the mRNA and protein levels were significantly increased at 5 and 10 μg/mL MDP (*p* < 0.05), with the highest mRNA expression observed at 10 μg/mL MDP (Fig. 2B).

Subsequently, we induced HCT116 cells with MDP (25 μg/mL) for different durations (0 h, 2 h, 4 h, 6 h, 8 h, 10 h), and detected the mRNA and protein expression levels of N4BP3. The results showed that, as the induction time increased, both the mRNA and protein levels of N4BP3 were increased (Fig. 2C, G); more specifically, the mRNA level was significantly increased at 2 h and 4 h (*p* < 0.05), with the highest expression observed at 4 h (Fig. 2C). Then, we induced HCT116 cells with MDP at different concentrations (0, 5, 15, 25, 50, 75 μg/mL) for 4 h, and detected the mRNA and protein expression levels of N4BP3. The results showed that, compared with MDP at 0 μg/mL, the mRNA and protein levels of N4BP3 were increased after induction with MDP at all other concentrations (Fig. 2D, H); more specifically the mRNA and protein level were significantly increased at 5, 15, 25, 50, and 75 μg/mL MDP (*p* < 0.05) (Fig. 2D).

Notably, the expression of N4BP3 was also found to be elevated in primary human peripheral blood mononuclear cells (PBMCs) induced by MDP (Fig. 2I, J). Specifically, the highest mRNA expression of N4BP3 was observed after 1 h of MDP induction at the concentration of 10 μg/mL, and the highest protein expression of N4BP3 was observed after 24 h of MDP induction at the concentration 10 μg/mL.

### N4BP3 knockdown downregulates the release of inflammatory factors

**Fig. 3:**
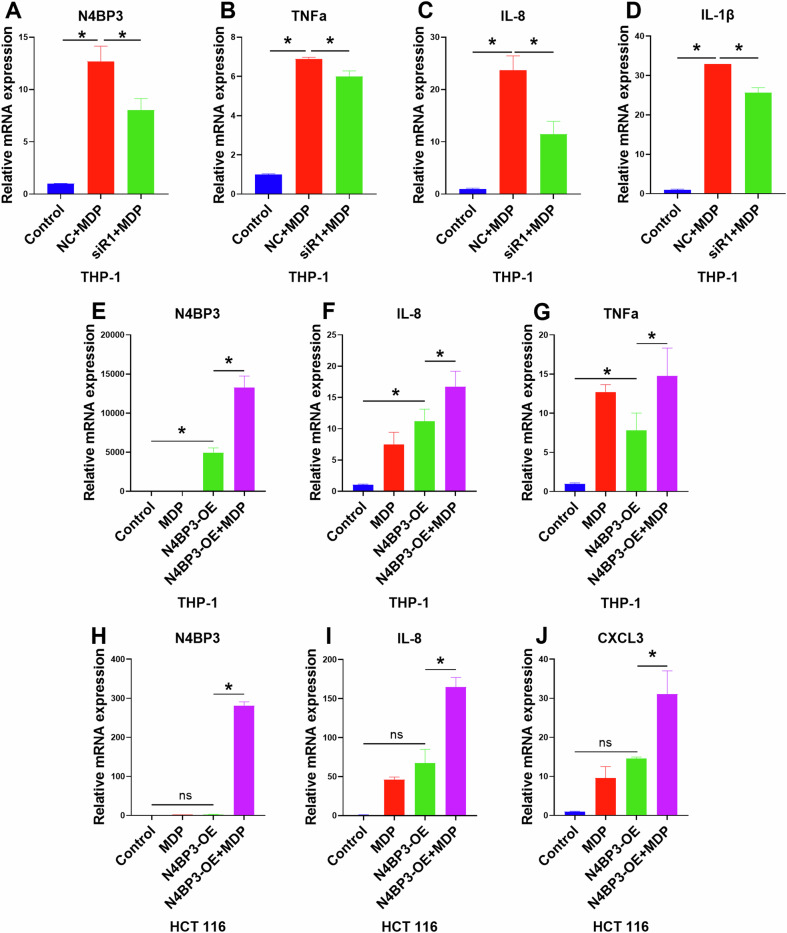
The mRNA expression of inflammatory factors induced by MDP in THP-1/HCT116 cells after N4BP3 knockdown/overexpression. **A** The expression of N4BP3 mRNA in THP-1 cells after N4BP3 knockdown. **B** The expression of TNFα mRNA in THP-1 cells after N4BP3 knockdown. **C** The expression of IL-8 mRNA in THP-1 cells after N4BP3 knockdown. **D** The expression of IL-1β mRNA in THP-1 cells after N4BP3 knockdown. **E** The expression of N4BP3 mRNA in THP-1 cells after N4BP3 overexpression. **F** The expression of IL-8 mRNA in THP-1 cells after N4BP3 overexpression. **G** The expression of TNFα mRNA in THP-1 cells after N4BP3 knockdown. **H** The expression of N4BP3 mRNA in HCT116 cells after N4BP3 overexpression. **I** The expression of IL-8 mRNA in HCT116 cells after N4BP3 overexpression. **J** The expression of CXCL3 mRNA in HCT116 cells after N4BP3 overexpression. *p < 0.05, indicates a statistically significant difference.

To investigate the effect of N4BP3 on inflammation, we prepared THP-1 cells with N4BP3 gene knockdown, and then induced the cells with MDP (10 μg/mL) for 3 h. Subsequently, the mRNA levels of inflammatory factors were detected through RT-qPCR. The results showed that after knocking down N4BP3 in THP-1 cells (Fig. 3A), the expression levels of TNFα, IL-8 and IL-1β induced by MDP were all significantly downregulated (*p* < 0.05) (Fig. 3B–D), indicating that N4BP3 knockdown can inhibit the production and release of inflammatory factors.

### N4BP3 overexpression promotes the release of inflammatory factors

We constructed THP-1 cells with N4BP3 gene overexpression and induced the cells with MDP (10 μg/mL) for 3 h. The mRNA levels of inflammatory factors were then detected through RT-qPCR. The results showed that after overexpressing N4BP3 in THP-1 cells (Fig. 3E), the mRNA expression levels of IL-8 and TNFα induced by MDP were significantly increased (*p* < 0.05) (Fig. 3F, G).

Further, we also constructed HCT116 cells with N4BP3 gene overexpression, and induced the cells with MDP (25 μg/mL) for 4 h. The mRNA levels of inflammatory factors were then detected through RT-qPCR. The results showed that after overexpressing N4BP3 in HCT116 cells (Fig. 3H), the mRNA expression levels of IL-8 and CXCL3 induced by MDP were significantly increased (*p* < 0.05) (Fig. 3I, J), indicating that N4BP3 overexpression can promote the production and release of inflammatory factors.

### N4BP3 activates the MAPK and NF-κB pathways

According to our experimental results, N4BP3 promotes the release of inflammatory factors by activating the NOD2 receptor pathway, which mainly functions through the downstream MAPK and NF-κB pathways. The key proteins of the MAPK pathway include ERK1/2 (ERK), JNK, and P38, while the key protein of the classical NF-κB pathway is NF-κB P65 (P65).

**Fig. 4:**
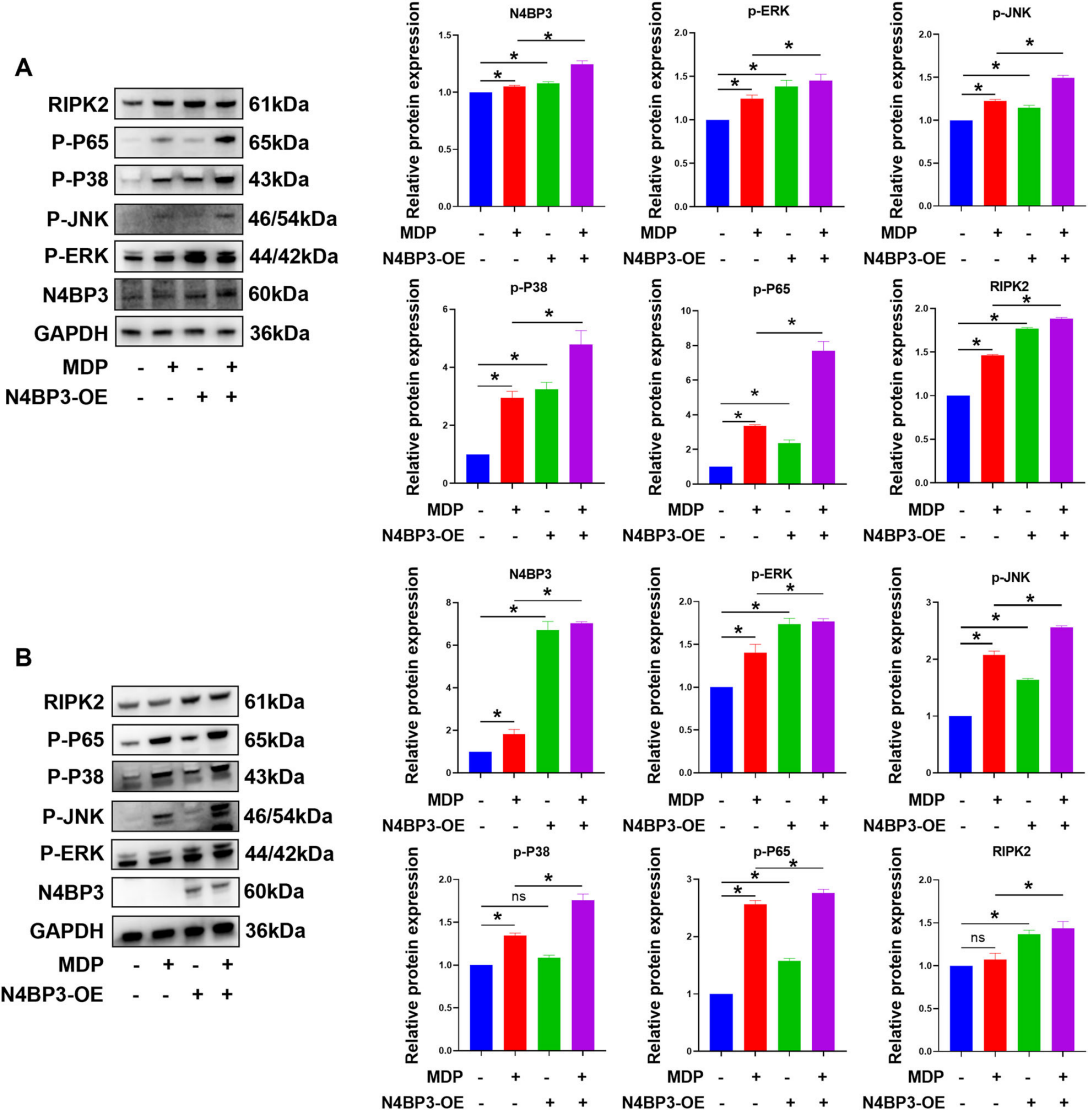
The expression levels of proteins induced by MDP in THP-1/HCT116 cells after N4BP3 overexpression. **A** The expression bands and relative of N4BP3, p-ERK1/2, p-JNK, p-P38, p-P65, and RIPK2 proteins induced by MDP (10 μg/mL) in THP-1 cells after N4BP3 overexpression. **B** The expression bands and relative of N4BP3, p-ERK1/2, p-JNK, p-P38, p-P65, and RIPK2 proteins induced by MDP (25 μg/mL) in HCT116 cells after N4BP3 overexpression. *p < 0.05, indicates a statistically significant difference.

To investigate the pathways through which N4BP3 promotes cellular inflammation, we constructed THP-1 cells with N4BP3 gene overexpression and induced the cells with MDP (10 μg/mL) for 3 h. Then, the expression levels of phospho (p)-ERK, p-JNK, p-P38, and p-P65 proteins were detected by Western blotting and the results showed that after overexpressing N4BP3 proteins in THP-1 cells, the expression levels of p-ERK, p-JNK, p-P38, and p-P65 proteins were all significantly increased (*p* < 0.05) (Fig. 4A).

We also construct HCT116 cells with N4BP3 gene overexpression and induced the cells with MDP (25 μg/mL) for 4 h. The expression levels of p-ERK, p-JNK, p-P38, and p-P65 proteins were detected by Western blotting and the results showed that after overexpressing N4BP3 proteins in HCT116 cells, the expression levels of p-ERK, p-JNK, p-P38, and p-P65 proteins were also significantly increased (*p* < 0.05) (Fig. 4B).

**Fig. 5:**
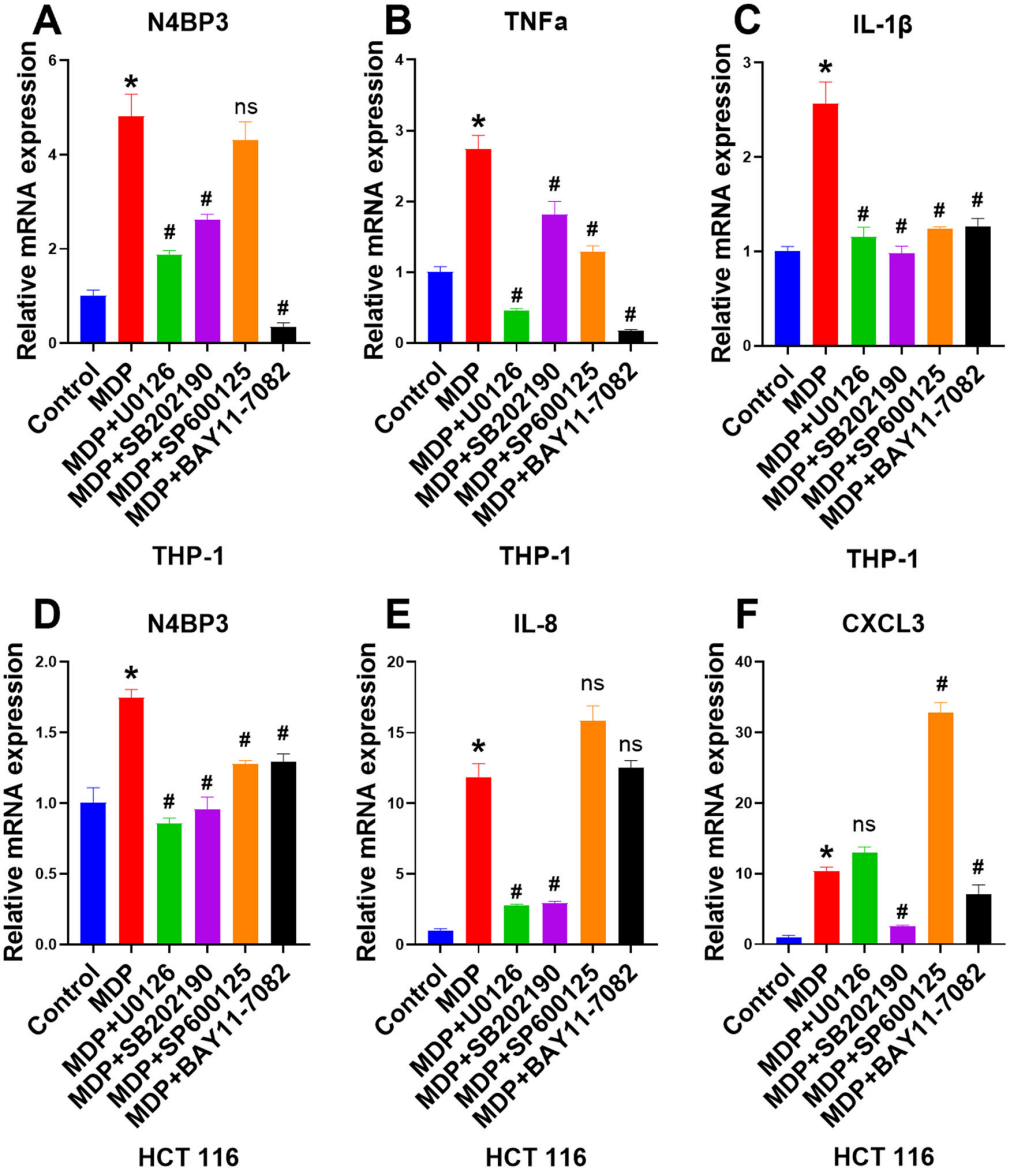
Expression of N4BP3 mRNA after inhibiting the MAPK and NF-κB pathways. **A**–**C** The expression of N4BP3, TNFα, IL-1β mRNA in THP-1 cells induced by MDP (10 μg/mL) after inhibiting ERK1/2, JNK, P38, and P65, respectively. **D**–**F** The expression of N4BP3 mRNA in HCT116 cells induced by MDP (25 μg/mL) after inhibiting ERK1/2, JNK, P38, and P65, respectively. *p < 0.05, indicates a statistically significant difference as compared to the control group. ^#^*p* < 0.05, indicates a statistically significant difference as compared to the MDP group.

Further, we induced both THP-1 cells and HCT116 cells with ERK, JNK, P38, and P65 inhibitors, i.e., U0126, SP600125, SB202190, and BAY11-7082, respectively, and detected the mRNA expression levels of N4BP3 and inflammatory factors. The results showed that after inhibiting ERK, JNK, P38, and P65, respectively, the mRNA expression levels of N4BP3 and inflammatory factors were all significantly reduced (Fig. 5), indicating that N4BP3 overexpression can activate both the MAPK pathway and NF-κB pathway.

Another interesting result is that N4BP3 overexpression in THP-1 cells can’t increase the expression of RIPK2 protein induced by Lipopolysaccharides (LPS) and Pam3csk4 but can activate the NF-κB pathways (Supplementally Fig. 1).

### N4BP3 interacts with RIPK2 and promotes its K63-linked ubiquitination

Since N4BP3 can simultaneously activate the MAPK pathway and NF-κB pathway, the sites of action of N4BP3 in the NOD2 pathway are likely to be the upstream signaling proteins in these two pathways. It has been pointed out that RIPK2 is the most common target protein in the NOD2 pathway. In this study, we would like to clarify whether N4BP3 also interacts with RIPK2 and promotes cellular inflammation through ubiquitination modification of RIPK2.

From the results above, it can be seen that N4BP3 overexpression in THP-1/HCT116 cells resulted in an increase in the expression of RIPK2 protein induced by MDP (Fig. 4A, B), suggesting that N4BP3 may activate the MAPK and NF-κB pathways through RIPK2.

**Fig. 6:**
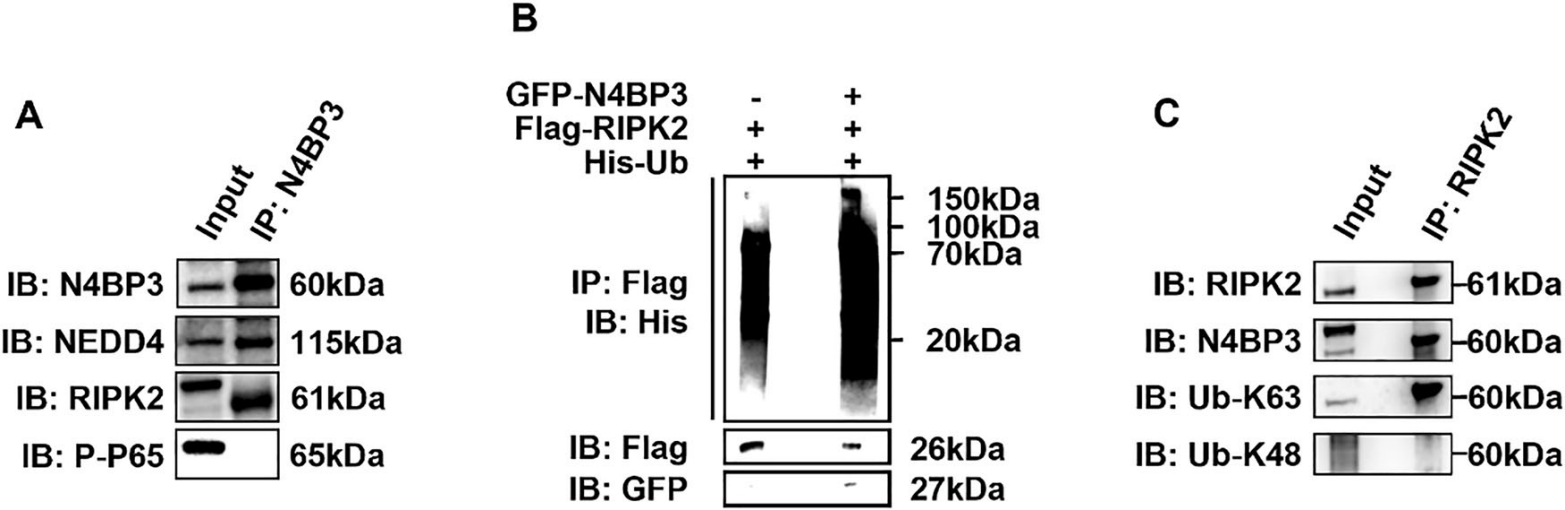
N4BP3 interacts with RIPK2 and promotes its ubiquitination. **A** After inducing HCT116 cells with MDP (25 μg/mL) for 4 h, immunoprecipitation of the anti-N4BP3 antibody can pull down N4BP3, NEDD4, and RIPK2, but not P-P65. **B** After co-transfecting GFP-N4BP3 plasmid, Flag-RIPK2 plasmid, and His-Ub plasmid and inducing HCT116 cells with MDP (25 μg/mL) for 4 h, immunoprecipitation of the anti-Flag antibody can pull down the His antibody for a larger range. **C** After inducing HCT116 cells with MDP (25 μg/mL) for 4 h, immunoprecipitation of the anti-RIPK2 antibody can pull down RIPK2, N4BP3, and Ub-K63, but not Ub-K48.

We constructed HCT116 cells with N4BP3 gene overexpression and induced the cells with MDP (25 μg/mL) for 4 h. Then, the proteins were extracted and added with the anti-N4BP3 antibody for immunoprecipitation, and the protein levels were detected by immunoblotting. The result showed that immunoprecipitation of RIPK2 could pull down N4BP3 and K63-linkage specific ubiquitin-linked N4BP3, but not K48-linkage specific ubiquitin-linked N4BP3 (Fig. 6A), confirming that N4BP3 is a connexin of NEDD4. Meanwhile, immunoprecipitation of N4BP3 can also pull down RIPK2 protein (Fig. 6A), indicating that N4BP3 has an interactive relationship with RIPK2.

Further, we co-transfected the GFP-N4BP3 plasmid, Flag-RIPK2 plasmid, and His-Ub plasmid into HCT116 cells as the experiment group and co-transfected the Flag-RIPK2 plasmid and His-Ub plasmid into HCT116 cells as the control group, for a comparative analysis. All the cells were induced by MDP (25 μg/mL) for 4 h, and the proteins were extracted and added with the anti-Flag antibody for immunoprecipitation assay. The expression levels of the Flag antibody, GFP antibody, and His antibody were then detected by immunoblotting. Compared with the control group, the expression of the His antibody was pulled down by a larger range after GFP-N4BP3 plasmid transfection and immunoprecipitation of the anti-Flag antibody (Fig. 6B), indicating that N4BP3 is able to promote the ubiquitination level of RIPK2.

In addition, we constructed HCT116 cells with N4BP3 gene overexpression, and induced the cells with MDP (25 μg/mL) for 4 h. Then, the proteins were extracted and added with the anti-RIPK2 antibody for immunoprecipitation, and the protein levels were detected by immunoblotting. The results showed that immunoprecipitation of RIPK2 could pull down the N4BP3 and Ub-K63 proteins, but not the Ub-K48 protein (Fig. 6C), indicating that N4BP3 is able to promote RIPK2 K63-linked ubiquitination.

### N4BP3 expression is upregulated in the CD colon tissue

**Fig. 7:**
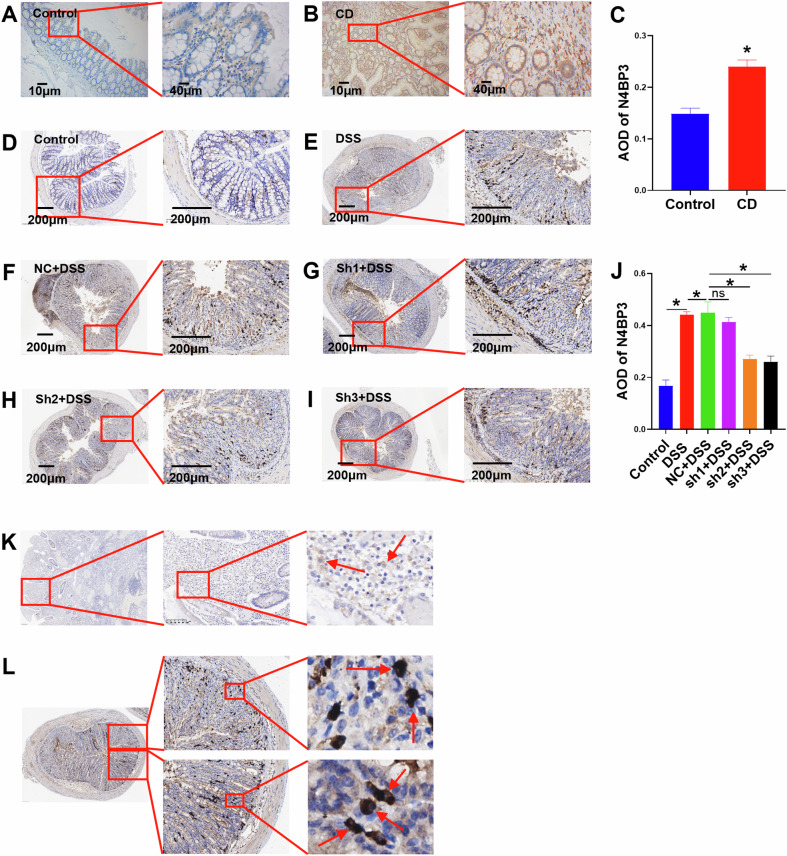
The expression of N4BP3 detected by IHC in the colon tissue from different human subjects and mouse groups. **A** The expression of N4BP3 in control colon tissue. **B** The expression of N4BP3 in CD colon tissue. **C** Quantitative analysis of N4BP3 expression in control colon tissue and CD colon tissue. **D**–**I** The expression of N4BP3 in the colon tissue of control group, DSS group, NC + DSS group, sh1 + DSS group, sh2 + DSS group, and sh3 + DSS group mice. **J** Quantitative analysis of N4BP3 expression in the colon tissue of mice from different groups. **K** Detection of cell types expressing N4BP3 in human colon samples by immunohistochemistry. **L** Detection of cell types expressing N4BP3 in murine samples by immunohistochemistry. *p < 0.05, indicates a statistically significant difference.

In order to detect the expression of N4BP3 in the human colon tissue, we collected 20 surgical pathological paraffin sections from CD patients and 20 normal control colon tissue from colon cancer patients who received surgery for tumor removal (sections taken at a distance of over 15 cm from the tumor edge), and then detected the expression of N4BP3 by IHC assay. The results showed that, compared with normal control, the expression of N4BP3 was significantly increased in the CD colon tissue (*p* < 0.05) (Fig. 7A–C); moreover, N4BP3 was predominantly expressed in macrophages, but not in epithelial cells, in the CD colon tissue (Fig. 7K).

### N4BP3 knockdown inhibits DSS-induced colitis in mice

To verify the pro-inflammatory effect of N4BP3 in IBD mice, we constructed mouse models with N4BP3 gene knockdown by injecting shRNA-N4BP3-AAV, and constructed IBD mice induced by 5% DSS to evaluate the Disease Activity Index (DAI) score. On the 6th day, the mice were dissected to measure the colon length, detect the N4BP3 expression in the colon tissue through IHC, and determine the pathological score of the colon tissue through HE staining.

According to the results, mice in the Control group showed the following features: no changes in mobility, glossy hair, normal eating and drinking behavior, no bloody stools around the anus, normal stool characteristics, negative occult blood, and weight gain.

**Fig. 8:**
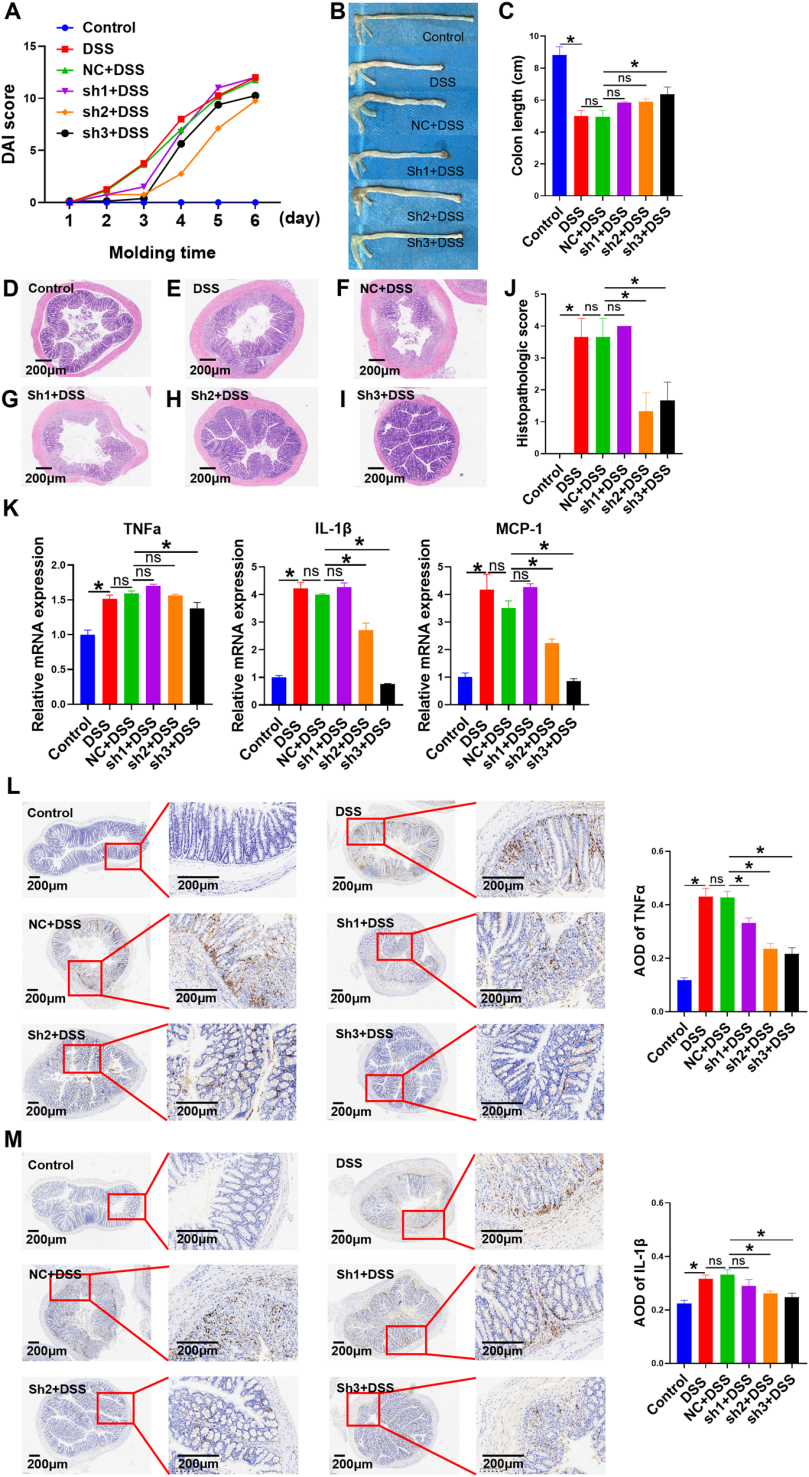
The DAI score, colon length, HE staining, and pathological score of mice in different groups. **A** Changes in DAI scores in mice from different treatment groups. **B**, **C** Comparison of colon length after dissection among mice from different treatment groups. **D**–**I** The HE stainings of colon tissue in control group, DSS group, NC + DSS group, sh1 + DSS group, sh2 + DSS group, and sh3 + DSS group mice. **J** Comparison of the pathological score of colon tissue among mice from different groups. **K** The expressions of TNFα, IL-1β and MCP-1 mRNAs in the colon tissue of control group, DSS group, NC + DSS group, sh1 + DSS group, sh2 + DSS group, and sh3 + DSS group mice. **L**, **M** The expressions of TNFα and IL-1β proteins in the colon tissue of control group, DSS group, NC + DSS group, sh1 + DSS group, sh2 + DSS group, and sh3 + DSS group mice. *p < 0.05, indicates a statistically significant difference.

Compared with Control mice, mice in the DSS group presented with the following changes: decreased mobility, dull hair, reduced food and water intake, bloody stools around the anus, positive occult blood, significant weight loss, significantly increased DAI score (*p* < 0.05) (Fig. 8A) (Supplementary Table 1), significantly shortened colon length (*p* < 0.05) (Fig. 8B, C), significantly increased N4BP3 expression (*p* < 0.05) (Fig. 7D, E, J), significantly increased histopathological score (*p* < 0.05) (Fig. 8D, E, J) (Supplementary Table 2), and significantly increased expression of inflammatory factors (*p* < 0.05) (Fig. 8K, L, M). The above results indicate successful modeling of IBD mice and that the N4BP3 expression was upregulated in the colon tissue of IBD mice. Moreover, N4BP3 was predominantly expressed in macrophages, but not in epithelial cells, in the colon tissue of IBD mice (Fig. 7L).

Compared with DSS mice, mice in the NC + DSS group, after fed with 5% DSS, showed no significant changes in mobility, hair glossiness, food and water intake, perianal blood test, occult blood test, weight loss rate, DAI score (*p* > 0.05) (Fig. 8A) (Supplementary Table 1), colon length (*p* > 0.05) (Fig. 8B, C), N4BP3 expression (*p* > 0.05) (Fig. 7E, F, J), histopathological score (*p* > 0.05) (Figs. 8E, F and 7J) (Supplementary Table 2), and the expression of inflammatory factors (*P* > 0.05) (Fig. 8K–M). The results suggest that the AAV used in our experiment did not significantly interfere with the experimental results data.

Compared with NC + DSS mice, mice in the sh2+DSS group, after fed with 5% DSS, showed the following changes: increased mobility, improved hair glossiness, increased food and water intake, reduced perianal bloody stool, lowered occult blood positivity, reduced weight loss, significantly reduced DAI score (*p* < 0.05) (Fig. 8A) (Supplementary Table 1), significantly increased colon length (*p* < 0.05) (Fig. 8B, C), significantly reduced N4BP3 expression (*p* < 0.05) (Fig. 7F, H, J), significantly reduced histopathological score (*p* < 0.05) (Fig. 8F, H, J) (Supplementary Table 2), and significantly reduced expression of inflammatory factors (*p* < 0.05) (Fig. 8K–M). In addition, compared with NC + DSS mice, mice in the sh3+DSS group presented with significantly reduced DAI score (*p* < 0.05) (Fig. 8A and Supplementary Table 1), significantly increased colon length (*p* < 0.05) (Fig. 8B, C), significantly reduced N4BP3 expression (*p* < 0.05) (Fig. 7F, I, J), significantly reduced histopathological score (*p* < 0.05) (Fig. 8F, I, J and Supplementary Table 2), and significantly reduced expression of inflammatory factors (*p* < 0.05) (Fig. 8K–M). The results indicate that N4BP3 knockdown in the colon tissue is able to reduce the degree of DSS-induced colitis in mice.

## Discussions

The pathogenesis of IBD has not yet been fully clarified, and the current treatment methods and efficacy for IBD are still subjected to limitations [30], making in-depth research on the mechanisms of IBD of great significance. At present, it is generally believed that the pathogenic mechanisms of IBD are related to the interaction between genetic, environmental, immune, and intestinal microbiota factors [31], where intestinal immune imbalance plays an important role [32]. The activation of intestinal immunity is usually mediated by PRRs, while the relationship between the NOD2 receptor and IBD is particularly important. There are currently several studies on the relationship between ubiquitination and NOD2, and the findings are summarized as follows: XIAP activates the NOD2 pathway by promoting the ubiquitination of RIPK2-K63 connection [33]; CYLD and OTULIN inhibit the NOD2 pathway by promoting the ubiquitination of RIPK2-M1 connection [34, 35]; cIAPs and Pellion3 activate the NOD2 pathway by promoting the ubiquitination of RIPK2-K63 connection [36, 37]; and TRAF4, TRIM27, and RNF34 inhibit the NOD2 pathway by promoting the ubiquitination of RIPK2-K48 connection [38–41]. Nonetheless, there are still many uncertainties to be explores in this field.

In our study, we screened out 19 ubiquitination-related genes with altered expression after MDP-induced NOD2 receptor activation by utilizing the transcriptomics method for the first time. We then quantitatively detected the mRNA expression of these 19 genes, of which N4BP3 presented with the most significant changes. Through multi-perspective verification with different cell types, different MDP induction durations, and different MDP concentrations, it was confirmed that activation of the NOD2 receptor can indeed upregulate the expression of N4BP3.

By constructing THP-1/HCT116 cells with N4BP3 knockdown or overexpression, we found that N4BP3 knockdown is able to reduce the mRNA expression of MDP-induced inflammatory factors, while N4BP3 overexpression has the opposite effect. This result emphasizes that N4BP3 plays a pro-inflammatory role in the body.

We then compared CD patients’ colon tissue with normal colon tissue and observed that the N4BP3 expression in the CD colon tissue was significantly higher than that in the normal tissue. By constructing IBD mouse models with DSS, we further found that the expression of N4BP3 in the colon tissue of IBD mice was also significantly higher than that in Control mice. Overall, both the cell and animal experiments suggest that N4BP3 plays a pro-inflammatory role in the body.

It has been shown that N4BP3 can promote the activation of NF-κB by promoting the interaction between MAVS and TRAF2 and the ubiquitination of TRAF2, leading to IFNβ production, which further promotes the RIG-I-MAVS signaling pathway. After overexpressing N4BP3, the ubiquitin ligase NEDD4 is recruited into MAVS, causing K63-linked polyubiquitination [25], indicating that the N4BP3-NEDD4 complex can potentially promote the RIG-I-MAVS mediated antiviral immunity. This process suggests that N4BP3 is likely to exert a protective effect in the body, which contradicts our results. Such consistency may be explained by the different pathways involved in the regulation mechanisms. The CARD domain at the N-terminus of NLR is able to bind and activate RIPK2 to phosphorylate IκB, thereby activating NF-κB, as well as TAK1 and MEKKs, leading to activation of the NF-κB pathway and MAPK pathway. The above process can further increase the mRNA expression of downstream inflammatory factors, which is of great significance in the occurrence and development of IBD [42]. We speculated that N4BP3 might promote the development of IBD by regulating the ubiquitination of RIPK2. To verify this speculation, we performed immunoprecipitation assay, and the results showed that N4BP3 is able to interact with the RIPK2 protein in the NOD2 pathway and promote the K63-linked ubiquitination of RIPK2 protein. Compared with the result that N4BP3 may promote mediated antiviral immunity, the ultimate effects revealed in our study are different, but all such effects are related to the process that N4BP3 interacts with a signaling protein in a certain pathway and promotes its ubiquitination, thereby promoting the activation of that signaling pathway.

Activation of RIPK2, as a downstream molecule of the NOD receptor and TOLL receptor, can promote the MAPK/NF-κB signaling pathway and to promote intestinal inflammation in IBD, whereas RIPK2 knockdown can attenuate this effect [43–45]. The autophagy-related protein 16 like 1 (ATG16L1) can bind to RIPK2 and downregulate its polyubiquitination, which inhibits the NF-κB pathway and suppresses the expression of inflammation factors [44]. In our study, it was found that N4BP3 binds to RIPK2 and upregulates its polyubiquitination, thereby promoting the MAPK and NF-κB pathways as well as promotes the expression of inflammation factors. These observations are consistent on the whole.

Our study provides a new perspective for explaining the pathogenesis of IBD. N4BP3, as a target gene and protein promoting IBD, may be utilized for the treatment of IBD by inhibiting its gene or protein.

## Material and methods

### Cell culture

The biomaterials used in this experiment include the human monocyte THP-1 (cultured using RPMI 1640) and the human colorectal cancer cell HCT116 (cultured using high-glucose DMEM). All cells were cultured in a carbon dioxide incubator at 37 °C and 5% CO_2_.

### RNA-seq

Add the cells into Trizol and mix thoroughly. Treat the cells with the following operations in sequence: total RNA extraction and quality inspection; mRNA enrichment; double-stranded cDNA synthesis; end repair and A-tailing, fragment selection, PCR enrichment, and library quality inspection. Perform sequencing using the Illumina sequencing platform.

### Cell siRNA transfection

Suspend the cells in 1 mL of double-antibody-free medium and then inoculate the cells into a 12-well cell culture plate at the density of 5 × 10^5^ to 1 × 10^6^ cells per well. Dilute 6 μL of 30x riboFET-CP Buffer to 1x using 54 μL of sterilized PBS solution. Add 5 μL of 20 μM siRNA (serial: GAGGGTACATGGACATGTA) storage solution and mix gently. Add 6 μL of riboFET-CP Reagent and mix gently. Incubate at room temperature for 15 min and add the solution to the cell suspension after the transfection complex stabilizes. Shake gently and place the cell culture plate in a carbon dioxide incubator for 48 h.

### Cell plasmid transfection

Suspend the cells in 2 mL of complete culture medium and then inoculate the cells into a 6-well cell culture plate at the density of 0.25 × 10^6^ to 1 × 10^6^ cells per well. Mix 125 μL of Opti MEM medium with 3.75 μL of Lipofectamine 3000 thoroughly and mix another 125 μL of Opti MEM medium with 2.5 μg of DNA and 5 μL of P3000 thoroughly. Mix the two solutions together and incubate at room temperature for 10–15 min. Add the mixture to the cell culture plate, shake gently, and place the cell culture plate in a carbon dioxide incubator for 48 h.

### Immunoprecipitation and Western blotting

Fully lyse the cells, take an appropriate amount of supernatant after high-speed centrifugation, add antibodies, and incubate on a flipping mixer. Wash the magnetic beads, add them into the antigen-antibody complex, and incubate on a flipping mixer. Wash the complex after incubation and add the SDS-PAGE loading buffer. Heat and cool the samples with a dry thermostat and use a magnetic rack to take the magnetic beads out. Collect the supernatant for SDS-PAGE.

Under the reducing condition, place the samples into the SDS-PAGE gel for electrophoresis, and then perform transmembrane with PVDF membrane. Incubate the samples with protein-free fast blocking solution for 15 min. Wash with the PBST solution for 10 min, and then incubate with the following antibodies: GAPDH (Abcam, ab128915), N4BP3 (Proteintech, 16733-1-AP), phospho (p)-ERK1/2 (CST, 4370), p-JNK (CST, 4668), p-P38 (CST, 4511), p-P65 (CST, 3033), RIPK2 (Proteintech, 15366-1-AP), NEDD4 (Proteintech, 21698-1-AP). Incubate at 4 °C, wash with the PBST solution three times, and further incubate with the PBST solution containing 2% milk for 50 min. Wash with the PBST solution three times and detect the expression levels using the chemiluminescence solution. Detect the optical density using ImageJ software. Incubate the PVDF membrane with the antibody stripping solution for 15 min. Wash the PVDF membrane with the PBST, and seal it properly for reuse.

### Real-time fluorescence quantitative PCR

Add Trizol and chloroform separately into the cells, and then centrifuge them at a high speed. Take the supernatant, add isopropanol and perform high-speed centrifugation. Aspirate and discard the supernatant, wash with anhydrous ethanol, and perform high-speed centrifugation. Aspirate and discard the supernatant, dilute the precipitate with sterile-and-enzyme-free water. Use cDNA Synthesis Kit to obtain cDNA. Add the cDNA, FastStart Essential DNA Green Master, and primers into an octuple tube in sequence. Place the tube into the fluorescence quantitative PCR instrument. See the primer sequence in Supplementary Table 3.

### Human specimens

We collected 20 surgical pathological paraffin sections from CD patients and 20 pathological paraffin sections of the normal colon tissue from colon cancer patients who received surgery for tumor removal (sections taken at a distance of over 15 cm from the tumor edge) during the period of 2012–2023 at University of South China Affiliated Changsha Central Hospital.

### Mouse grouping

C57BL/6 mice were divided into 6 groups: 1) Control group: free to drink and eat throughout the entire process; 2) DSS group: free to drink 5% DSS; 3) NC + DSS group: free to drink 5% DSS after intraperitoneal injection of Negative control (NC) adeno-associated virus (AAV); 4) sh1 + DSS group: free to drink 5% DSS after intraperitoneal injection of N4BP3 knockdown sequence 1 AAV (shRNA1-N4BP3-AAV, sh1); 5) sh2 + DSS group: free to drink 5% DSS after intraperitoneal injection of sh2; 6) sh3 + DSS group: free to drink 5% DSS after intraperitoneal injection of sh3.

### Construction of N4BP3 knockout mice and the related IBD mouse models

Intraperitoneally inject the N4BP3 knockdown AAV into the mice, (one AAV injection for each mouse only). The mice were allowed to drink distilled water freely for 3 weeks, and then were fed with DSS solution. See the N4BP3 knockdown sequence in Supplementary Table 4.

### Disease activity index (DAI) scoring

Starting from the first day when the mice began to drink 5% DSS, measure their body weight, observe their stool conditions (including whether there are bloody stools), and test for positive occult blood on a daily basis. See scoring criteria in Supplementary Table 5.

### Hematoxylin-eosin (HE) staining

Embed the colon tissue with paraffin to create test sections. Place the sections in a 55 °C oven for 30 min. Dewax the sections with xylene, and then soak the sections in ethanol and distilled water for hydration. Add hematoxylin, 1% hydrochloric acid ethanol, and blue buffer dropwise into the sections for hematoxylin staining. Soak the sections in ethanol at different concentrations for eosin staining. Soak the sections in anhydrous ethanol and xylene at different concentrations. Perform air drying, seal the sections, and observe the images.

### Colon histopathological scoring

Prepare colon tissue sections for HE staining. Observe the inflammatory cell infiltration and glandular damage under a microscope and perform histopathological scoring of the colon. See scoring criteria in Supplementary Table 6.

### Immunohistochemistry (IHC) staining

Embed the colon tissue with paraffin to create test sections. Place the sections in a 55 °C oven for 30 min. Soak the sections in xylene for dewaxing, and then soak them in anhydrous ethanol and distilled water for hydration. Soak the sections in 3% H_2_O_2_ for inactivation. Soak the sections in the antigen repair solution and heat them using a microwave for antigen repair. Add blocking buffer dropwise for blocking, and then add the antibody dropwise for incubation. Wash the sections with the PBST solution. Add the DBA coloring solution dropwise for color development, and then add double distilled water dropwise to terminate color development. Add hematoxylin dropwise for re-staining, and then rinse the sections with running tap water for blueing. Soak the sections in ethanol at different concentrations sequentially for dehydration. Add neutral gum dropwise, ensure ventilation, and observe the images after sealing.

### Data analysis and statistical considerations

Statistical data analysis and plotting were conducted using GraphPad Prism 8.0.1 software. The independent sample *t*-test was applied for comparisons between two groups. The one-way ANOVA test was applied for comparisons among three or more groups. *p* < 0.05 was considered statistically significant. The immunoblotting protein bands and IHC staining images were semi-quantitatively detected using KF-Viewer and Image J.

## Supplementary information

Supplementary Figure

Supplementary Table

Dataset 1

## Data Availability

All data and material are included in this manuscript.
