# Correction: N4BP3 facilitates NOD2-MAPK/NF-κB pathway in inflammatory bowel disease through mediating K63-linked RIPK2 ubiquitination

**DOI:** 10.1038/s41420-025-02574-x

**Published:** 2025-08-26

**Authors:** Wang Jiang, Yan Zhao, Min Han, Jiafan Xu, Kun Chen, Yi Liang, Jie Yin, Jinyue Hu, Yueming Shen

**Affiliations:** 1 Department of Digestive Diseases, The Affiliated Changsha Central Hospital, Hengyang Medical School, University of South China, 161 Shaoshan Road, Changsha, 410000 China; 2 Department of Pathology, The Affiliated Changsha Central Hospital, Hengyang Medical School, University of South China, 161 Shaoshan Road, Changsha, 410000 China; 3 Department of Cardiovascular Diseases, The Affiliated Changsha Central Hospital, Hengyang Medical School, University of South China, 161 Shaoshan Road, Changsha, 410000 China; 4 Department of Orthopaedics, The Affiliated Changsha Central Hospital, Hengyang Medical School, University of South China, Changsha, Hunan China; 5 Department of Gastrointestinal Surgery, The Second Affiliated Hospital, Hengyang Medical School, University of South China, 35 Jiefang Road, Hengyang, 421000 China; 6 Medical Research Center, The Affiliated Changsha Central Hospital, Hengyang Medical School, University of South China, Changsha, 410004 China

**Keywords:** NOD-like receptors, Inflammatory bowel disease, NOD-like receptors

Correction to: *Cell Death Discovery* 10.1038/s41420-024-02213-x, published online 17 October 2024

First, our description of the sentence “The results showed that immunoprecipitation of RIPK2 could pull down the N4BP3 and Ub-K63 proteins, but not the Ub-K48 protein” is inaccurate. And it should be revised to “The result showed that immunoprecipitation of RIPK2 could pull down N4BP3 and K63-linkage specific ubiquitin-linked N4BP3, but not K48-linkage specific ubiquitin-linked N4BP3”.

For the molecular weights of Ub-K63 and Ub-K48 in Figure 6C and RIPK2 in Figure 6A, our markings do need to be changed, and we have uploaded both the revised figure 6 and original full length western blots of figure 6 in the attachment.

Second, we apologize that we mischaracterized some words and misquoted a reference in the discussion section.

“XIAP activates the NOD2 pathway by promoting the ubiquitination of RIPK2-M1 connection” should be modified to “XIAP activates the NOD2 pathway by promoting the ubiquitination of RIPK2-K63 connection”.

RNF31 does activate the NF-κB pathway through M1-linked ubiquitination, so RNF31 should be excluded from “and TRAF4, RNF31, TRIM27, and RNF34 inhibit the NOD2 pathway by promoting the ubiquitination of RIPK2-K48 connection [38,39,40,41]”.

Original full length western blots of figure 6.
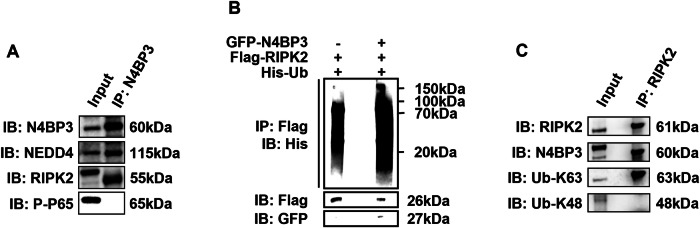


**Figure 6. N4BP3 interacts with RIPK2 and promotes its ubiquitination**. [**A**] After inducing HCT116 cells with MDP (25 μg/mL) for 4h, immunoprecipitation of the anti-N4BP3 antibody can pull down N4BP3, NEDD4, and RIPK2, but not P-P65. [**B**] After co-transfecting GFP-N4BP3 plasmid, Flag-RIPK2 plasmid, and His-Ub plasmid and inducing HCT116 cells with MDP (25 μg/mL) for 4h, immunoprecipitation of the anti-Flag antibody can pull down the His antibody for a larger range. [**C**] After inducing HCT116 cells with MDP (25 μg/mL) for 4h, immunoprecipitation of the anti-RIPK2 antibody can pull down RIPK2, N4BP3, and K63-linkage specific ubiquitin-linked N4BP3, but not K48-linkage specific ubiquitin-linked N4BP3.
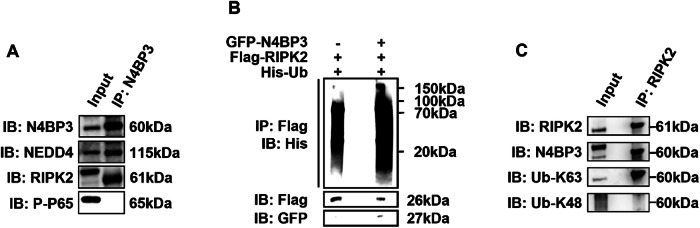


The original article has been corrected.

