# Supplementary material for: N4BP3 facilitates NOD2-MAPK/NF-κB pathway in inflammatory bowel disease through mediating K63-linked RIPK2 ubiquitination
Source: Cell Death Discov. 2024 Oct 17;10:440. doi: 10.1038/s41420-024-02213-x (PMC11487068; doi:10.1038/s41420-024-02213-x)
Supplement: Supplementary file 1 — Supplementary Figure [file 41420_2024_2213_MOESM1_ESM.doc]

**N4BP3 facilitates NOD2 - MAPK / NF-κB pathway in inflammatory bowel disease through mediating K63-linked RIPK2 ubiquitination**

**Wang Jiang^1*^, Yan Zhao^2*^, Min Han^3^, Jiafan Xu^1^, Kun Chen^4^, Yi Liang^5^, Jie Yin^1^, Jinyue Hu^6^, Yueming Shen^1#^**


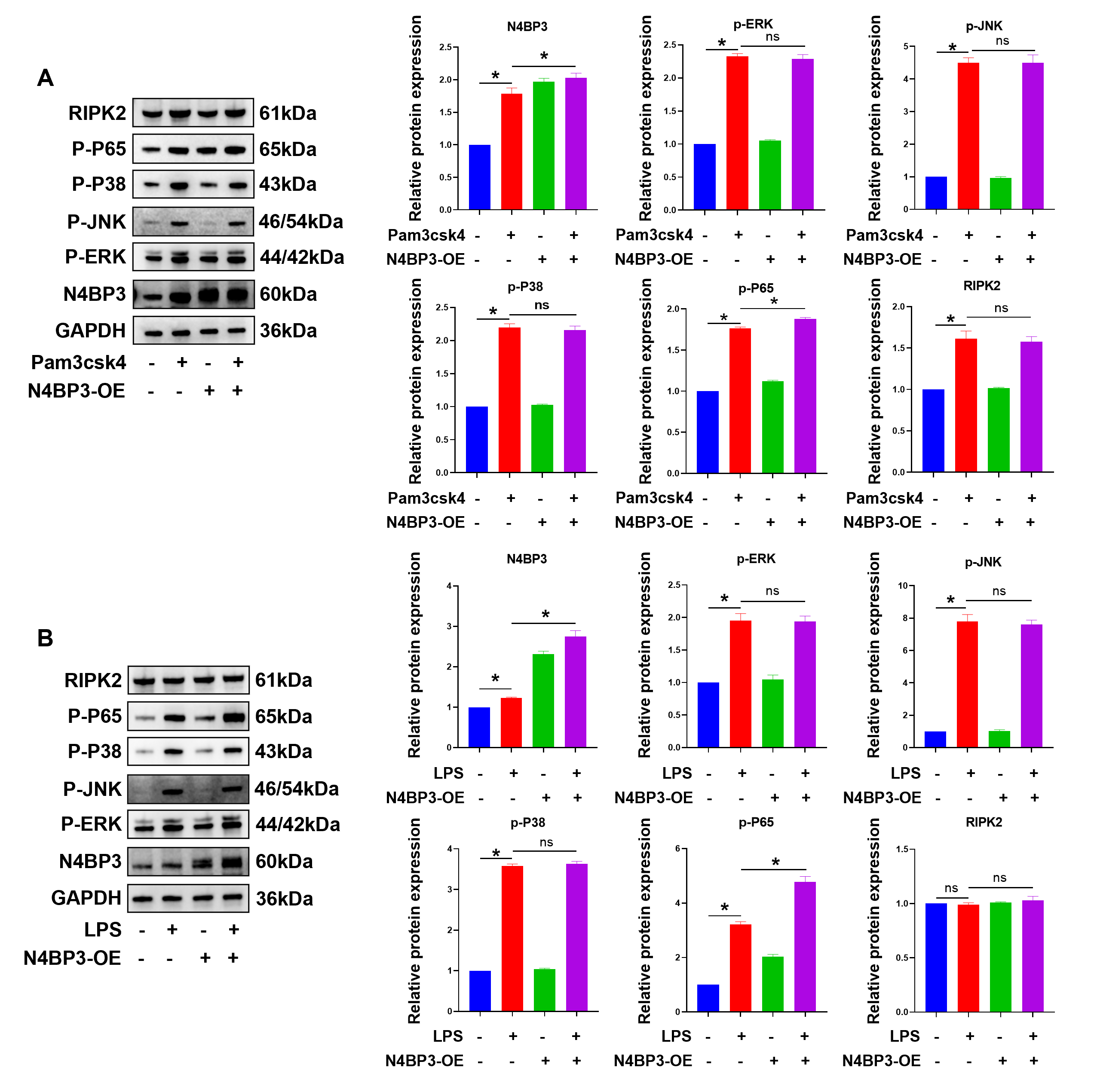
**Supplementary Figure 1. The expression levels of proteins induced by Pam3csk4 / LPS in THP-1 cells after N4BP3 overexpression.** [A] The expression bands and relative of N4BP3, p-ERK1/2, p-JNK, p-P38, p-P65, and RIPK2 proteins induced by Pam3csk4 (200ng/mL) in THP-1 cells after N4BP3 overexpression. [B] The expression bands and relative of N4BP3, p-ERK1/2, p-JNK, p-P38, p-P65, and RIPK2 proteins induced by LPS (1μg/mL) in THP-1 cells after N4BP3 overexpression. * *P*<0.05, indicates a statistically significant difference.
