# Supplementary material for: N4BP3 facilitates NOD2-MAPK/NF-κB pathway in inflammatory bowel disease through mediating K63-linked RIPK2 ubiquitination
Source: Cell Death Discov. 2024 Oct 17;10:440. doi: 10.1038/s41420-024-02213-x (PMC11487068; doi:10.1038/s41420-024-02213-x)
Supplement: Supplementary file 2 — Supplementary Table [file 41420_2024_2213_MOESM2_ESM.docx]

**N4BP3 facilitates NOD2 - MAPK / NF-κB pathway in inflammatory bowel disease through mediating K63-linked RIPK2 ubiquitination**

**Wang Jiang^1*^, Yan Zhao^2*^, Min Han^3^, Jiafan Xu^1^, Kun Chen^4^, Yi Liang^5^, Jie Yin^1^, Jinyue Hu^6^, Yueming Shen^1#^**

**Supplementary Table 1. DAI scores**

| Molding-Time | Control | DSS | NC+DSS | sh1+DSS | sh2+DSS | sh3+DSS |
| --- | --- | --- | --- | --- | --- | --- |
| Day 1 | 0.00 | 0.00 | 0.00 | 0.00 | 0.00 | 0.13 |
| Day 2 | 0.00 | 1.25^*^ | 1.13 | 0.75 | 1.75 | 0.13 |
| Day 3 | 0.00 | 3.75^*^ | 3.63 | 1.50^#^ | 0.75^#^ | 0.38^#^ |
| Day 4 | 0.00 | 8.00^*^ | 7.00 | 6.75 | 2.75^#^ | 5.63 |
| Day 5 | 0.00 | 10.25^*^ | 10.13 | 11.00 | 7.13^#^ | 9.38 |
| Day 6 | 0.00 | 12.00^*^ | 11.75 | 12.00 | 11.00^#^ | 10.25^#^ |

^*^ *P*<0.05, indicates a statistically significant difference as compared to the control group. ^#^ *P*<0.05, indicates a statistically significant difference as compared to the NC+DSS group.

**Supplementary Table 2. Histopathologic scores of the colon**

| clusters | Histopathology score |
| --- | --- |
| Control | 0.00 |
| DSS | 3.67^*^ |
| NC+DSS | 3.67 |
| sh1+DSS | 4.00 |
| sh2+DSS | 1.33^#^ |
| sh3+DSS | 1.67^#^ |

^*^ *P*<0.05, indicates a statistically significant difference as compared to the control group. ^#^ *P*<0.05, indicates a statistically significant difference as compared to the NC+DSS group.

**Supplementary Table 3. Primer sequences**

| Primer name | Primer Sequences |
| --- | --- |
| GAPDH Forward primer (5'→3') | AATCCCATCACCATCTTCCA |
| GAPDH Reverse primer (5'→3') | CCTGCTTCACCACCTTCTTG |
| N4BP3 Forward primer (5'→3') | AACGAGCCTGCCGACTATG |
| N4BP3 Reverse primer (5'→3') | ACTTTGCATGGATAGGAAGCC |
| TRIM36 Forward primer (5'→3') | GAGCTGTTTACCCACCCATTG |
| TRIM36 Reverse primer (5'→3') | CTGATCCCACATCGTTGAATGA |
| TRIM13 Forward primer (5'→3') | ACTTTGATACCAGTCAGTGGGAAGA |
| TRIM13 Reverse primer (5'→3') | AGACAATGACAAGGCCAAGCA |
| RNF144B Forward primer (5'→3') | GAGCACCGAGCCCTCTTTGGGA |
| RNF144B Reverse primer (5'→3') | GAGCGCAGCCTTCATTGCGT |
| CBX4 Forward primer (5'→3') | AGTGGAGTATCTGGTGAAATGGA |
| CBX4 Reverse primer (5'→3') | TCCTGCCTTTCCCTGTTCTG |
| CBX8 Forward primer (5'→3') | GTGAAATGGAAGGGATG |
| CBX8 Reverse primer (5'→3') | GTTTTGGGCTTGGGTC |

Supplementary table 3 continued

| Primer name | Primer Sequences |
| --- | --- |
| RNF122 Forward primer (5'→3') | GCACAGAGTGAGCGATACGG |
| RNF122 Reverse primer (5'→3') | GGAGCACGCCTAACTCATCC |
| TIFA Forward primer (5'→3') | CAAACAGGTTTCCCGAGTTCA |
| TIFA Reverse primer (5'→3') | TGTCCACGATCAGATTGGTCTT |
| ZC3H12A Forward primer (5'→3') | TTGTGAAGCTGGCCTACGAG |
| ZC3H12A Reverse primer (5'→3') | TCAGGGGGCATAAACTTGTCA |
| USP2 Forward primer (5'→3') | GGCTCGTTGTGCATTGCAGT |
| USP2 Reverse primer (5'→3') | GGCAGGACTGGAGACC |
| KLHL21 Forward primer (5'→3') | GACTGCGACTCTAAACGGACTC |
| KLHL21 Reverse primer (5'→3') | ATGTATTGTCGTATCCCCCAGA |
| DTX2 Forward primer (5'→3') | ACTGCAACGGCAATAAGGA |
| DTX2 Reverse primer (5'→3') | GGGAGCGACATCTGGAAC |
| TRAF3 Forward primer (5'→3') | TCTTGAGGAAAGACCTGCGAG |
| TRAF3 Reverse primer (5'→3') | GCGATCATCGGAACCTGACT |
| TNFAIP3 Forward primer (5'→3') | AGAGCAACTGAGATCGAGCCA |
| TNFAIP3 Reverse primer (5'→3') | CTGGTTGGGATGCTGACACTC |
| BIRC3 Forward primer (5'→3') | AAGCTACCTCTCAGCCTACTTT |
| BIRC3 Reverse primer (5'→3') | CCACTGTTTTCTGTACCCGGA |
| UBE2B Forward primer (5'→3') | TCCTTCAGAATCGATGGAGTCCA |
| UBE2B Reverse primer (5'→3') | GCTGGACTGTTAGGATTCGGTTC |
| CYLD Forward primer (5'→3') | CTTGCCTGACTGGGACT |
| CYLD Reverse primer (5'→3') | TTCTGACCACCATCTCG |
| MINDY3 Forward primer (5'→3') | TCCGAACTGACTAAAGAGCTGA |
| MINDY3 Reverse primer (5'→3') | CAGGTGCAATAACAGCACAGG |
| TIFA Forward primer (5'→3') | CAAACAGGTTTCCCGAGTTCA |
| TIFA Reverse primer (5'→3') | TGTCCACGATCAGATTGGTCTT |
| TNFα Forward primer (5'→3') | AAGCCTGTAGCCCATGTTGT |
| TNFα Reverse primer (5'→3') | AGTCGGTCACCCTTCTCCA |
| IL-8 Forward primer (5'→3') | TTGGCAGCCTTCCTGATTT |
| IL-8 Reverse primer (5'→3') | TCAAAAACTTCTCCACAACCC |
| IL-1β Forward primer (5'→3') | GACGGACCCCAAAAGATGAA |
| IL-1β Reverse primer (5'→3') | CAGCCACGAGGCTTTTTGTT |

**Supplementary Table 4. AAV-shRNA-N4BP3 sequences**

| AAV-shRNA-N4BP3 | sequences |
| --- | --- |
| negative control AAV (NC) | **Top strand:**  GATCCGTTCTCCGAACGTGTCACGTAATTCAAGAGATTACGTGACACGTTCGGAGAATTTTTTC  **Bottom strand:**  AATTGAAAAAATTCTCCGAACGTGTCACGTAATCTCTTGAATTACGTGACACGTTCGGAGAACG |
| shRNA1-N4BP3-AAV (sh1) | **Top strand:**  AATTCGACCAGGATGTTTCTAACTCCTTTACTCGAGTAAAGGAGTTAGAAACATCCTGGTCTTTTTTG  **Bottom strand:**  GATCCAAAAAAGACCAGGATGTTTCTAACTCCTTTACTCGAGTAAAGGAGTTAGAAACATCCTGGTCG |
| shRNA2-N4BP3-AAV (sh2) | **Top strand:**  AATTCGACGACTTCATGAGGTGGCCCAGAAACTCGAGTTTCTGGGCCACCTCATGAAGTCGTTTTTTTG  **Bottom strand:**  GATCCAAAAAAACGACTTCATGAGGTGGCCCAGAAACTCGAGTTTCTGGGCCACCTCATGAAGTCGTCG |
| shRNA3-N4BP3-AAV (sh3) | **Top strand:**  AATTCGCCGCGGAGATCAGTCTGTTGAAACACTCGAGTGTTTCAACAGACTGATCTCCGCGGTTTTTTG  **Bottom strand:**  GATCCAAAAAACCGCGGAGATCAGTCTGTTGAAACACTCGAGTGTTTCAACAGACTGATCTCCGCGGCG |

**Supplementary Table 5. Disease Activity Index (DAI) Scoring Scale for Mice***

| Weight loss rate（%） | stool properties ^#^ | Occult blood or blood in the stool | score |
| --- | --- | --- | --- |
| ≤1 | normal stool | negativity | 0 |
| 1-5 | semi-formed stool | negativity | 1 |
| 5-10 | semi-formed stool | positivity | 2 |
| 10-15 | watery stool | positivity | 3 |
| ≥15 | watery | having blood in one's stool | 4 |

*DAI score = weight loss rate score + stool characterization score + occult blood or blood in stool score, weight loss rate = (pre-experimental body weight - post-modeling body weight)/pre-experimental body weight × 100%, with a minimum of 0 and a maximum of 12 total points. #Normal stools were formed stools with rounded stripes; semi-formed stools were formed, thin, soft stools that did not stick to the anus; thin, soft stools were unformed stools that stuck to the anus; and watery leaks were watery stools that stuck to the anus.

**Supplementary Table 6. Histopathological scoring criteria for the colon***

| pathological changes | | score |
| --- | --- | --- |
| inflammatory cell infiltration | Glandular damage |  |
| No inflammatory cell infiltration | Normal morphology of intestinal epithelium | 0 |
| Inflammatory cell infiltration involving the mucosal layer | Partial glandular damage | 1 |
| Inflammatory cell infiltration involving the submucosa | Extensive glandular damage | 2 |
| Inflammatory cell infiltration involving the muscularis propria | Partial loss of glandular crypts | 3 |
| Inflammatory cell infiltration involving the entire intestinal wall | Loss of large glandular crypts | 4 |

*Colonic Histopathology Score = Inflammatory Cell Infiltration Score + Glandular Damage Score with a minimum total score of 0 and a maximum score of 8.
