# Supplementary figures and images for: N4BP3 facilitates NOD2-MAPK/NF-κB pathway in inflammatory bowel disease through mediating K63-linked RIPK2 ubiquitination
Source: Cell Death Discov. 2024 Oct 17;10:440. doi: 10.1038/s41420-024-02213-x (PMC11487068; doi:10.1038/s41420-024-02213-x)

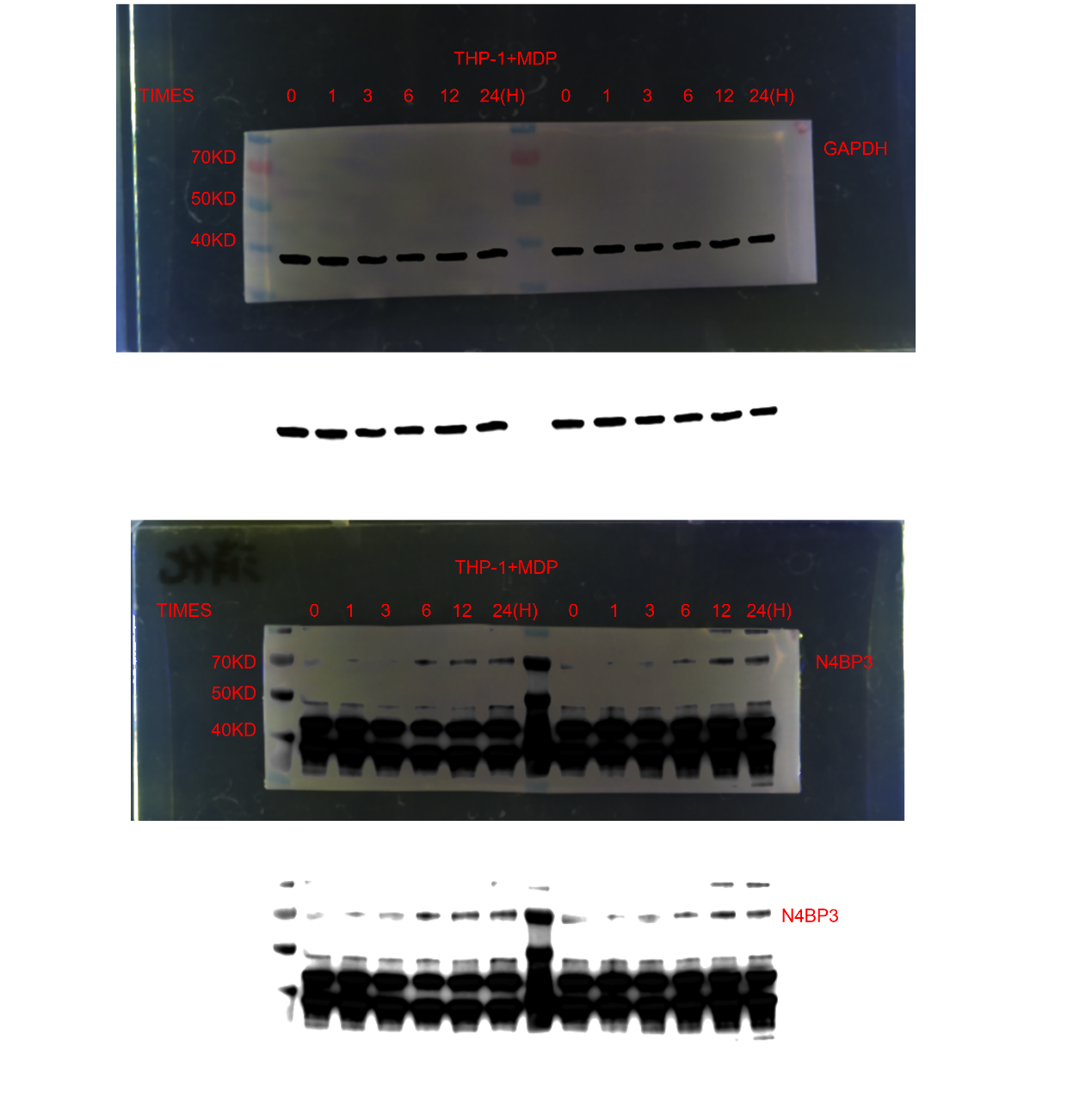


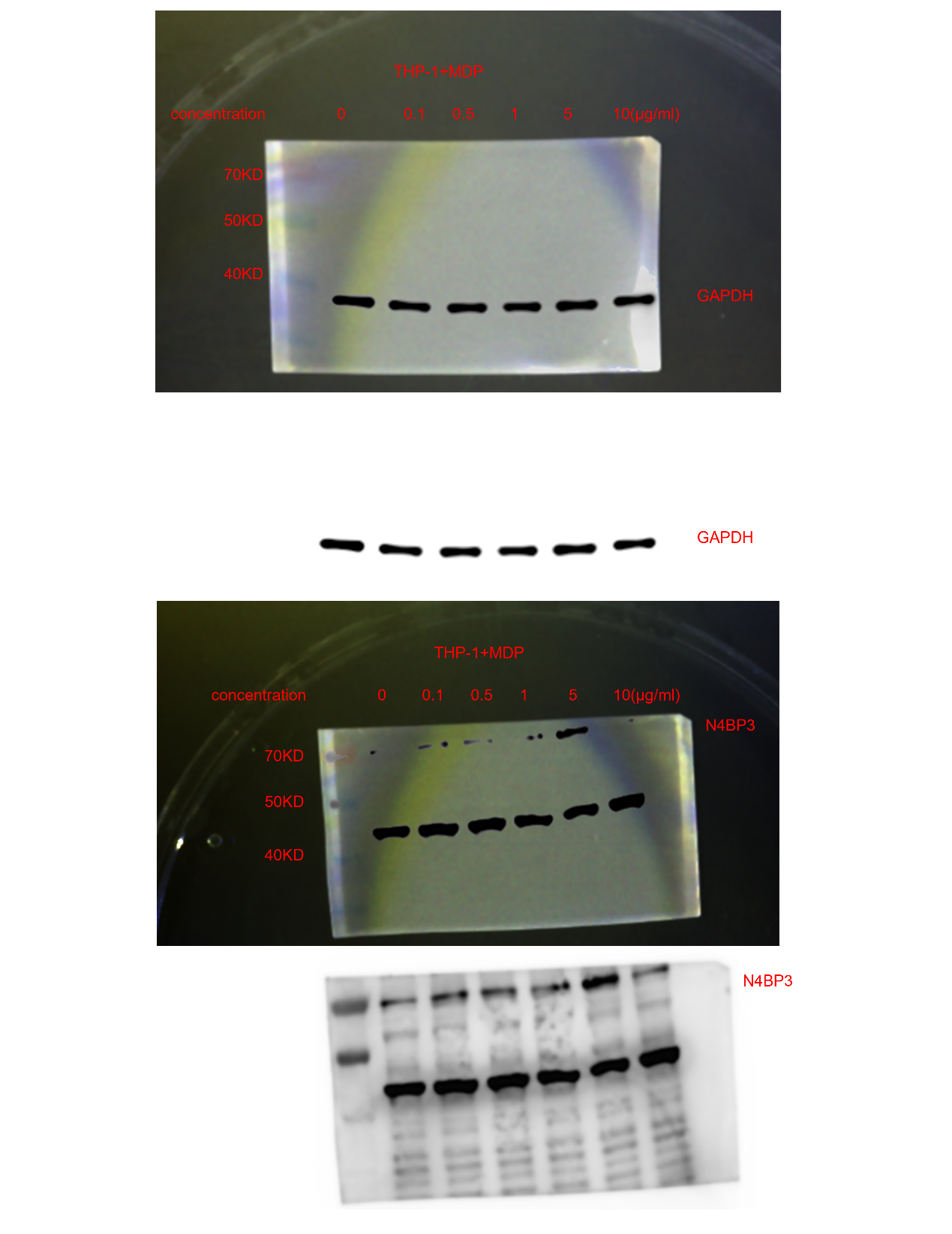


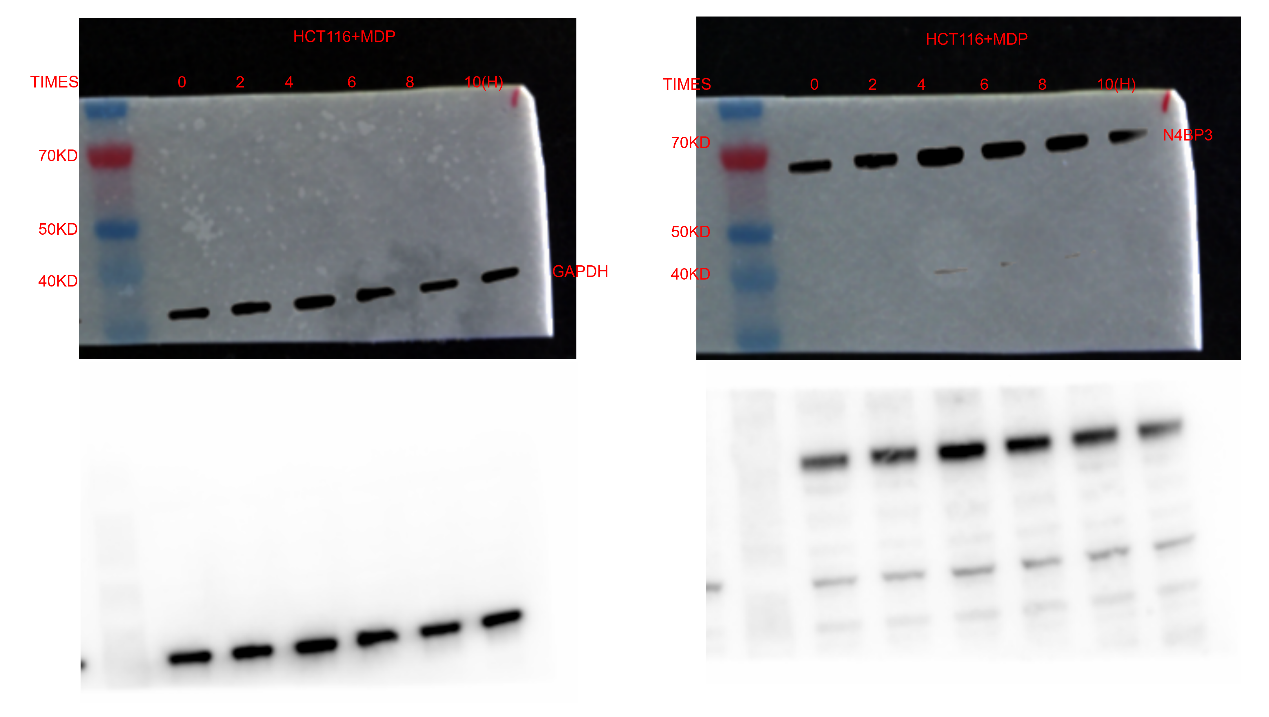


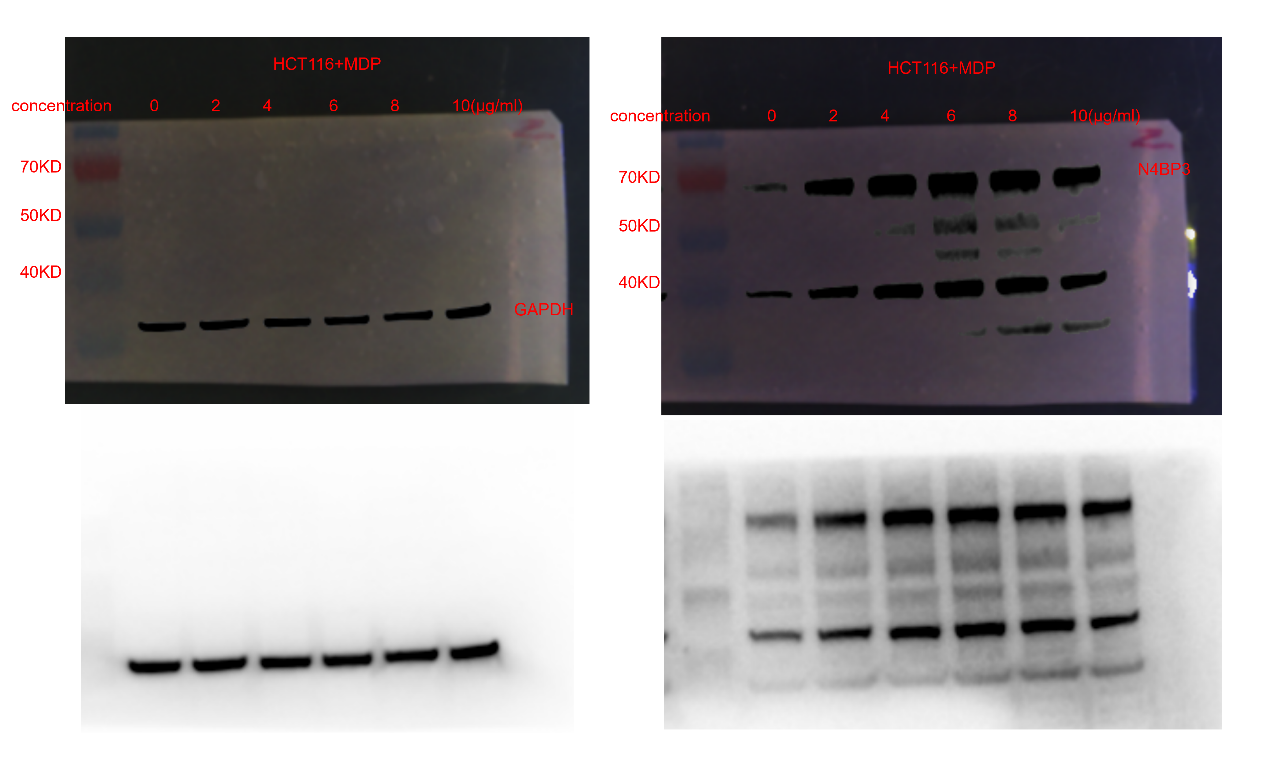


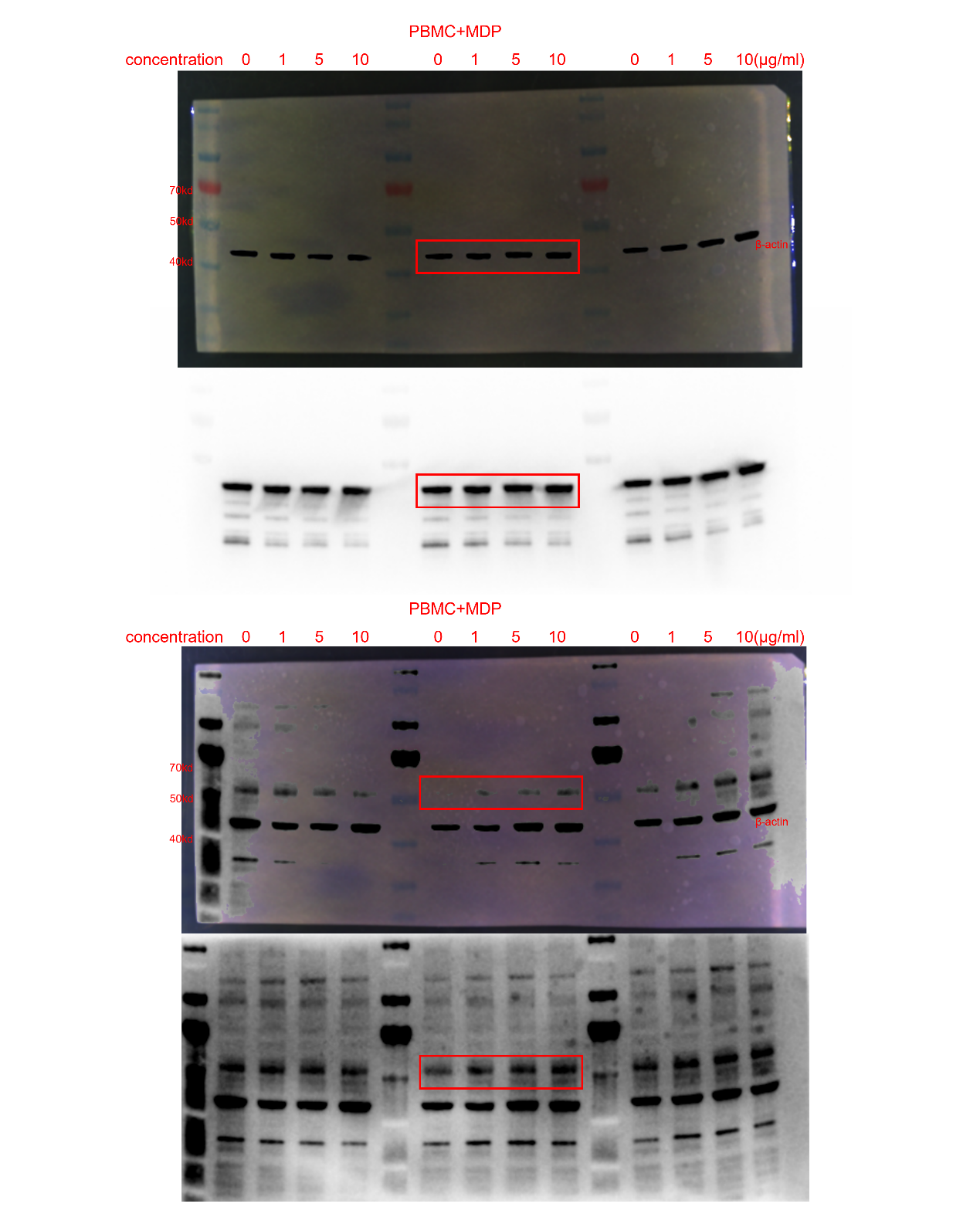


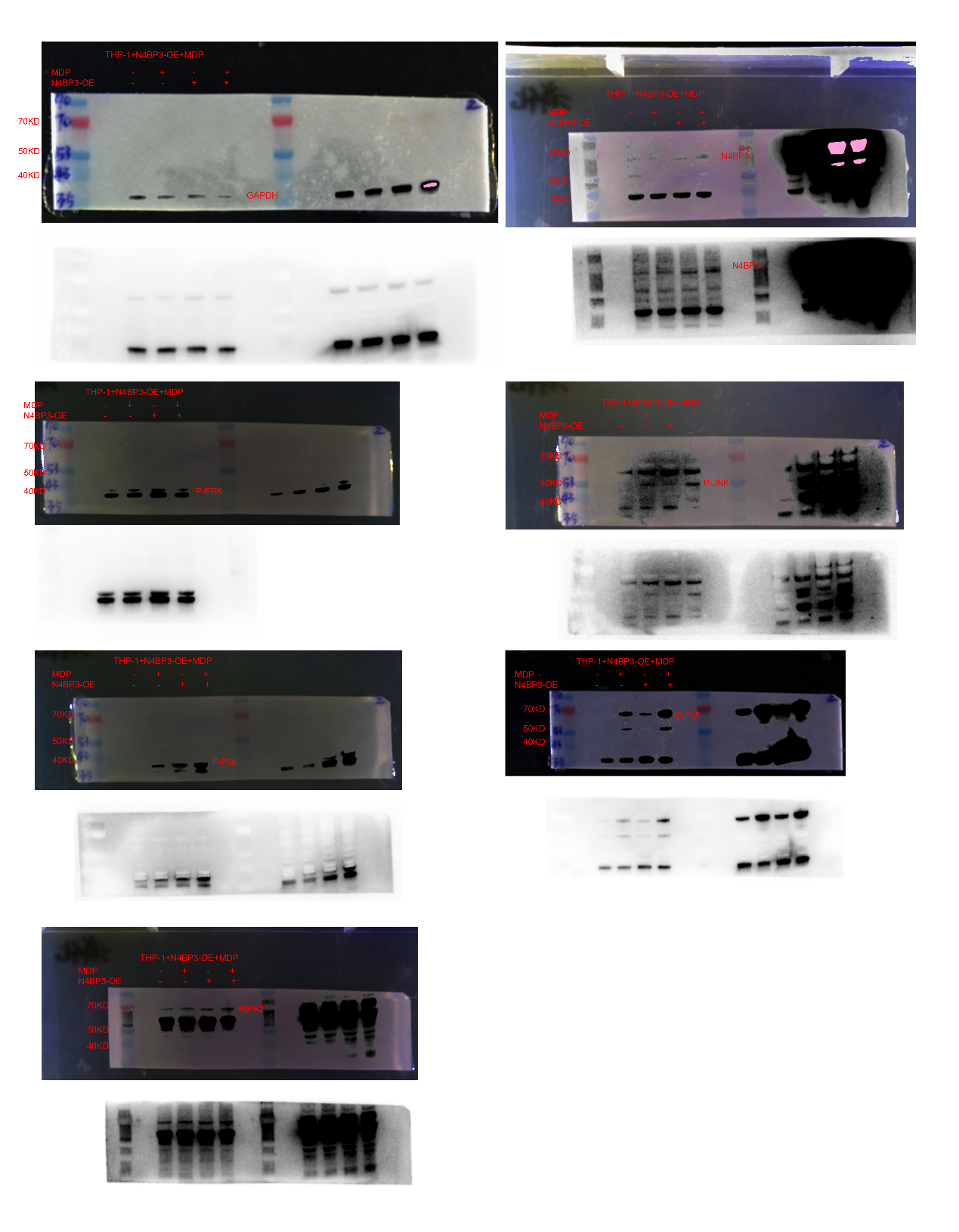


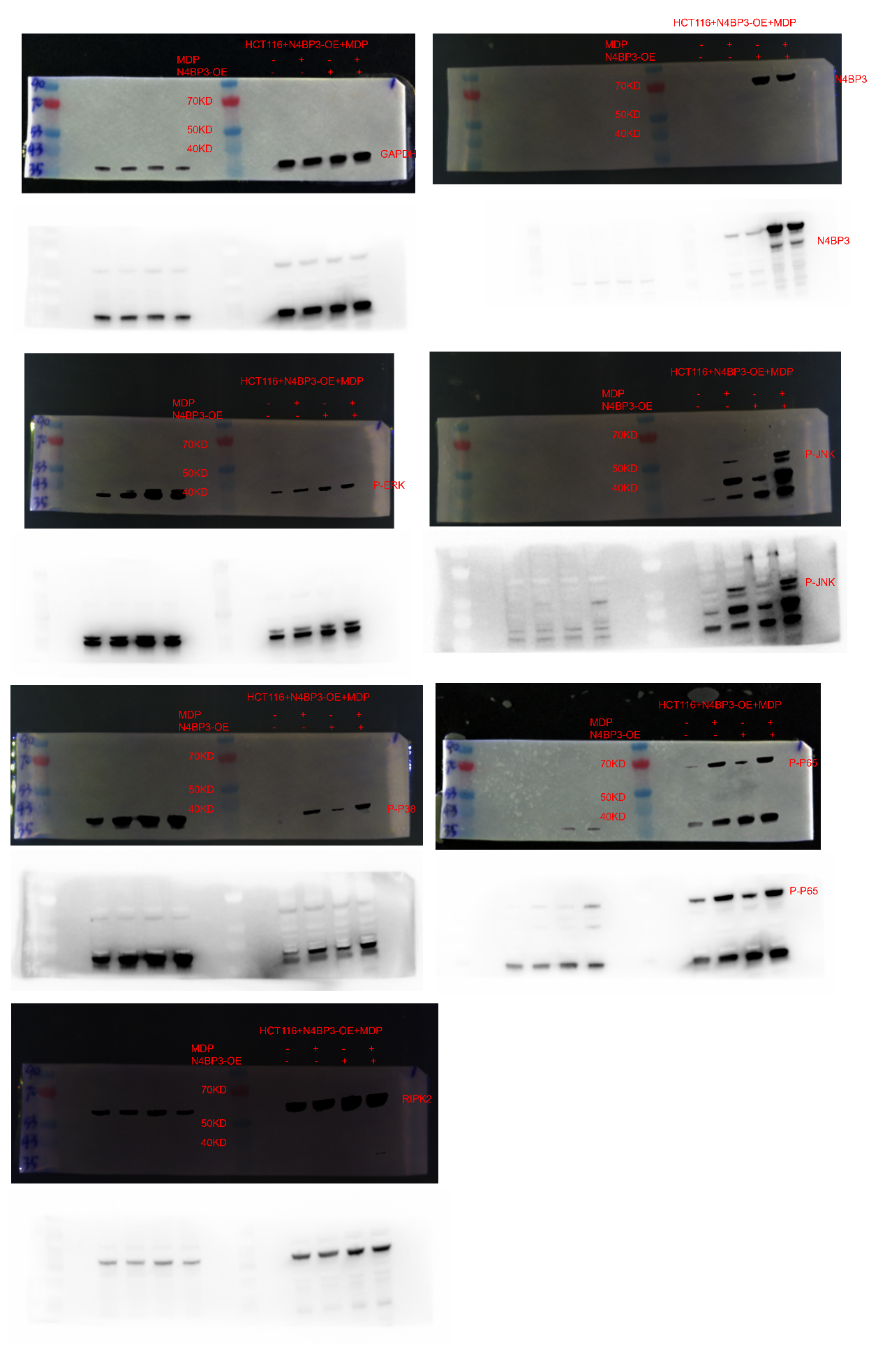


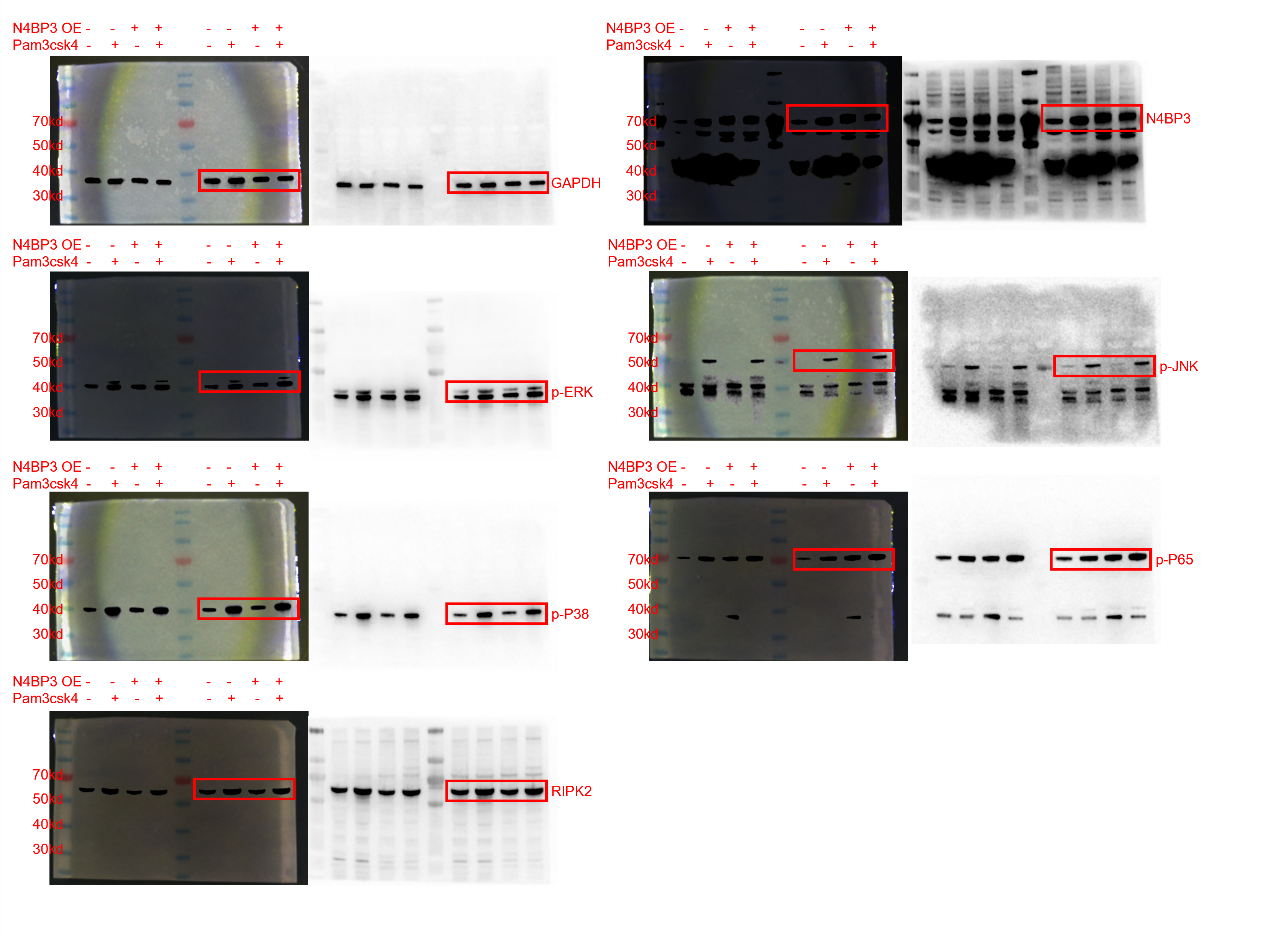


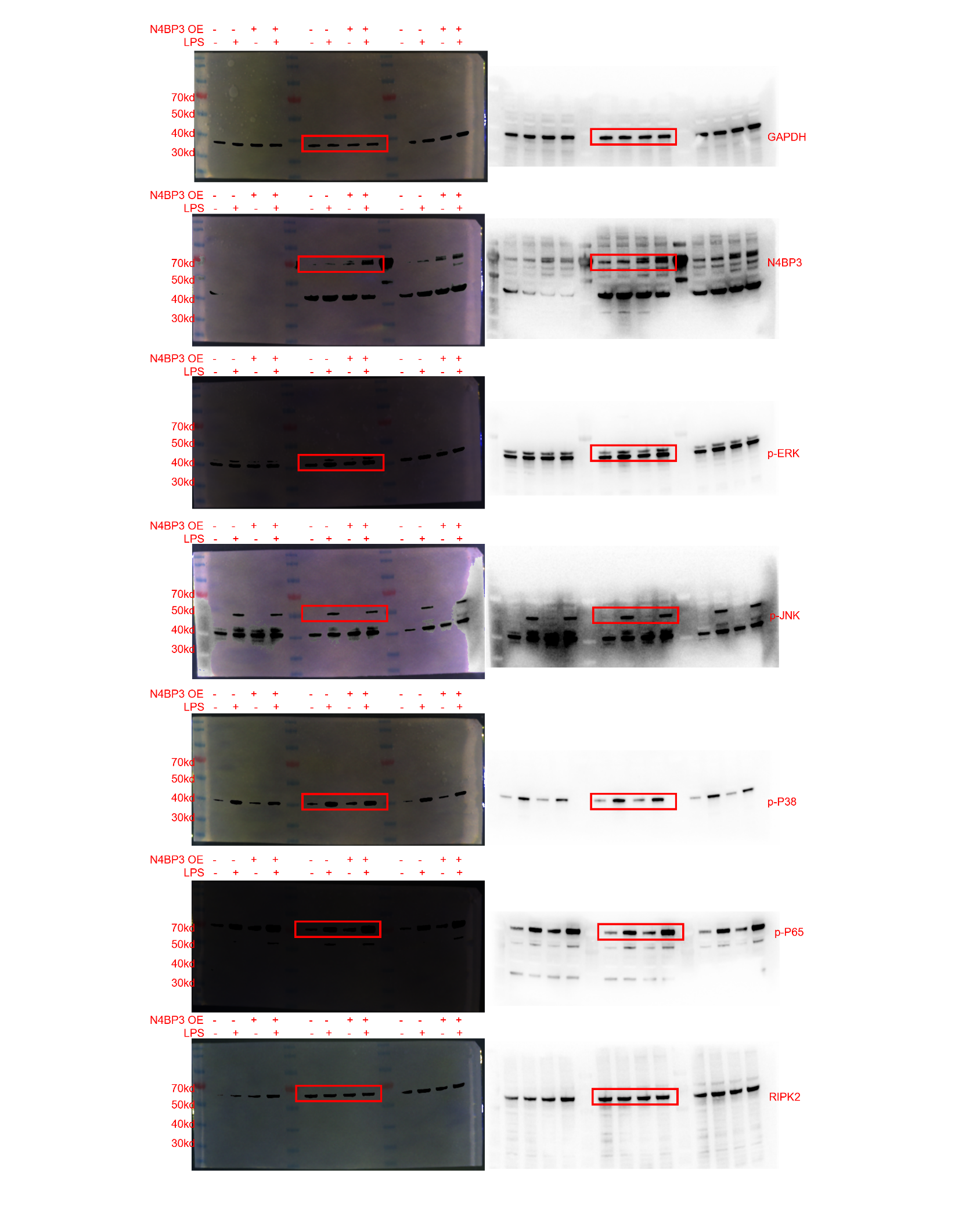


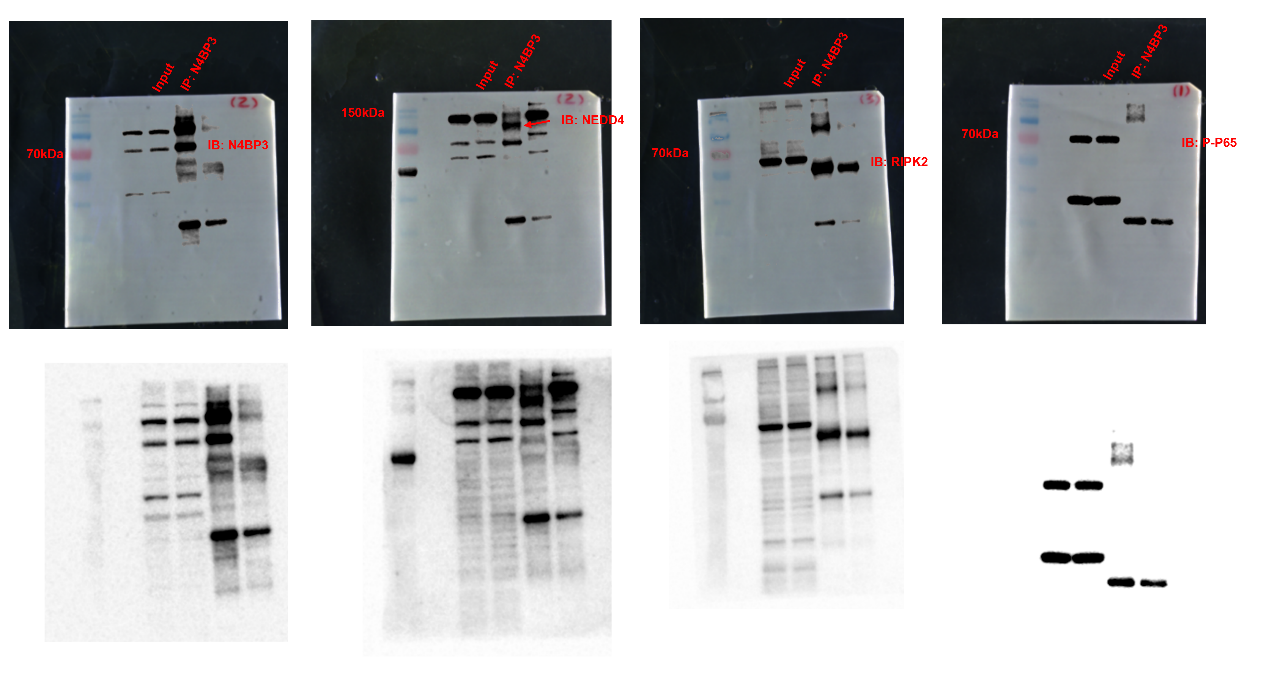


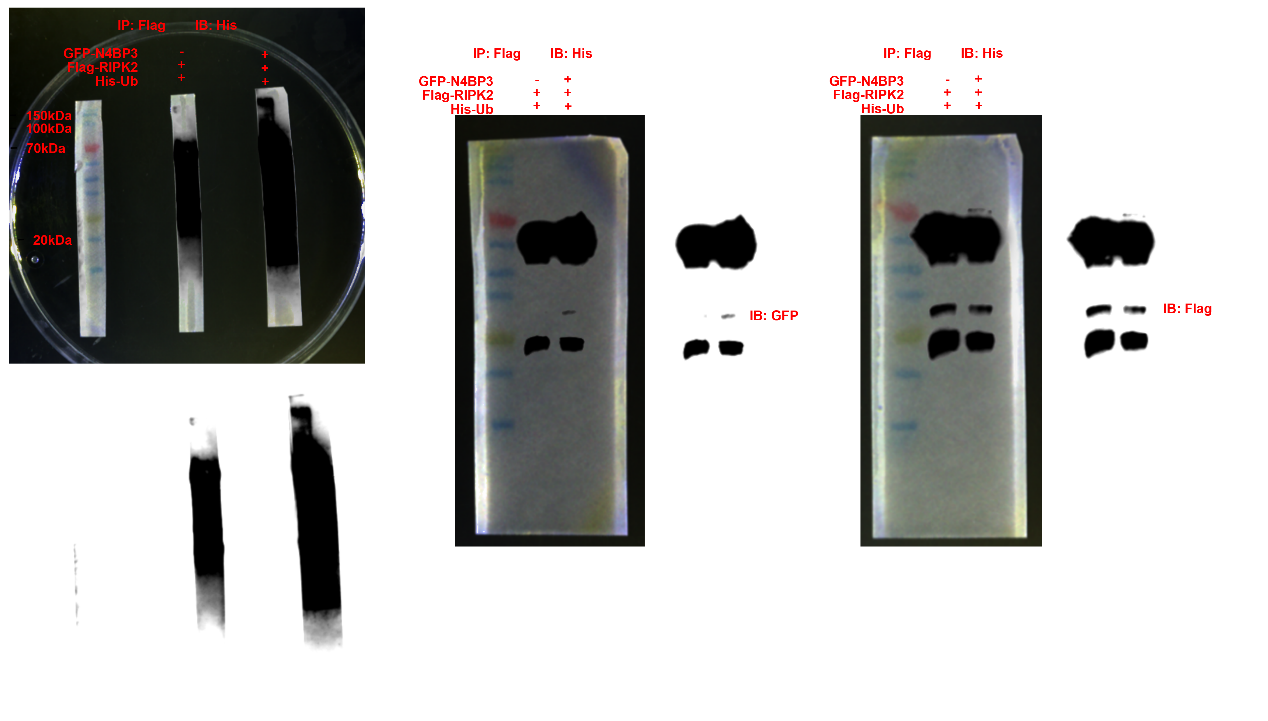


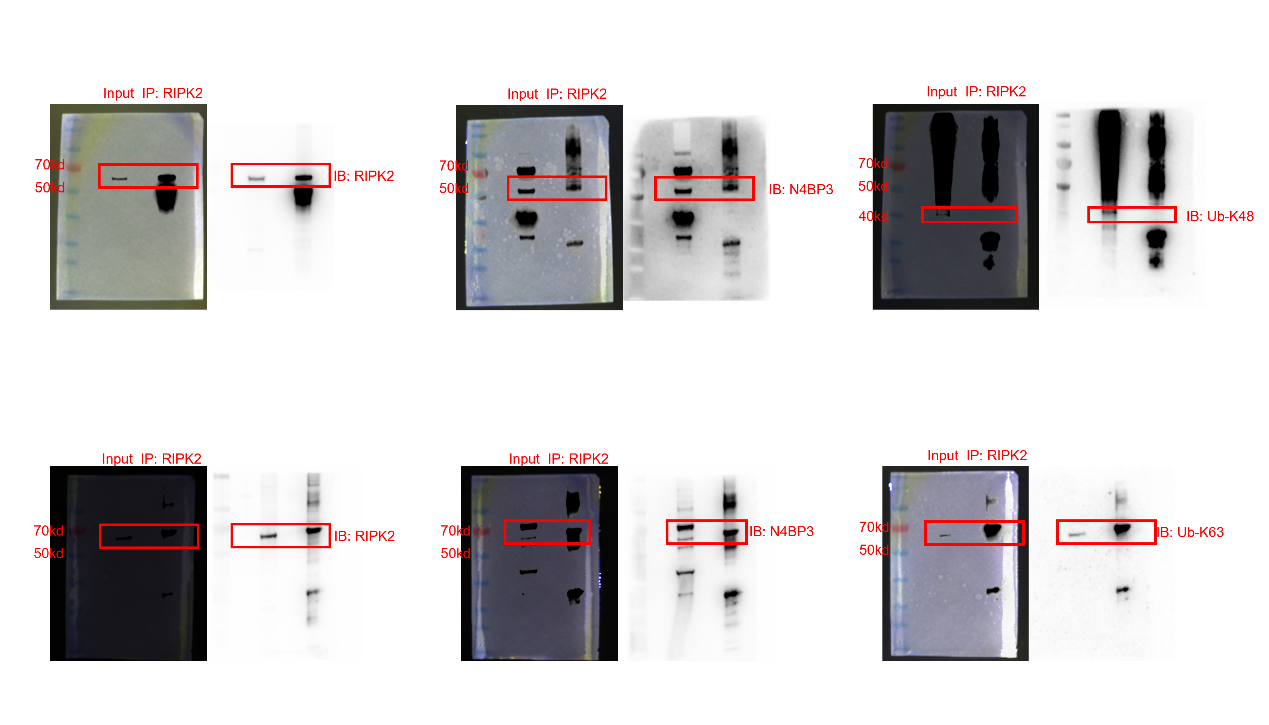

Supplement: Supplementary file 3 — Dataset 1 [file 41420_2024_2213_MOESM3_ESM.docx]
